# Electrolyte Engineering Toward High Performance High Nickel (Ni ≥ 80%) Lithium‐Ion Batteries

**DOI:** 10.1002/advs.202305753

**Published:** 2023-12-03

**Authors:** Tiantian Dong, Shenghang Zhang, Zhongqin Ren, Lang Huang, Gaojie Xu, Tao Liu, Shitao Wang, Guanglei Cui

**Affiliations:** ^1^ Qingdao Industrial Energy Storage Research Institute Qingdao Institute of Bioenergy and Bioprocess Technology Chinese Academy of Sciences Qingdao 266101 China; ^2^ Shandong Energy Institute Qingdao 266101 China; ^3^ Qingdao New Energy Shandong Laboratory Qingdao 266101 China

**Keywords:** electrolyte engineering, functional additive, high nickel lithium‐ion battery, solvent, thermal runaway mechanism

## Abstract

High nickel (Ni ≥ 80%) lithium‐ion batteries (LIBs) with high specific energy are one of the most important technical routes to resolve the growing endurance anxieties. However, because of their extremely aggressive chemistries, high‐Ni (Ni ≥ 80%) LIBs suffer from poor cycle life and safety performance, which hinder their large‐scale commercial applications. Among varied strategies, electrolyte engineering is very powerful to simultaneously enhance the cycle life and safety of high‐Ni (Ni ≥ 80%) LIBs. In this review, the pivotal challenges faced by high‐Ni oxide cathodes and conventional LiPF_6_‐carbonate‐based electrolytes are comprehensively summarized. Then, the functional additives design guidelines for LiPF_6_‐carbonate ‐based electrolytes and the design principles of high voltage resistance/high safety novel electrolytes are systematically elaborated to resolve these pivotal challenges. Moreover, the proposed thermal runaway mechanisms of high‐Ni (Ni ≥ 80%) LIBs are also reviewed to provide useful perspectives for the design of high‐safety electrolytes. Finally, the potential research directions of electrolyte engineering toward high‐performance high‐Ni (Ni ≥ 80%) LIBs are provided. This review will have an important impact on electrolyte innovation as well as the commercial evolution of high‐Ni (Ni ≥ 80%) LIBs, and also will be significant to breakthrough the energy density ceiling of LIBs.

## Graphical abstract

Electrolyte engineering is powerful in enhancing the cycle life and safety of high‐Ni LIBs. In this review, the pivotal challenges faced by high‐Ni oxide cathodes and conventional LiPF_6_‐carbonate‐based electrolytes are summarized. To resolve these challenges, the functional additives design guidelines for LiPF_6_‐carbonate‐based electrolytes and the design principles of high voltage resistance/high safety novel electrolytes are elaborated.

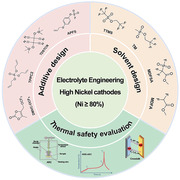

## 1 Introduction

With the global fossil energy crisis, environmental pollution, and increasing greenhouse gas CO_2_ emissions, the efficient use of renewable energy (such as solar, wind, hydro, tidal, biomass, etc.) is attracting great attention. The intermittent and unstable intrinsic nature of renewable energy sources makes the development of static energy storage systems (ESSs) very crucial. Rechargeable lithium‐ion batteries (LIBs) have become a very important energy storage technology because of their advantages like high energy density, long cycle life, low self‐discharging, wide operating temperature range, and rapid charging/discharging capability, etc.^[^ ^1^, ^2^ ^]^ Since the successful commercialization of LIBs by Sony Corporation in 1991, their applications have rapidly expanded from portable electronics to electric vehicles (EVs) and renewable ESSs.^[^ ^3^ ^]^ The 2019 Noble Prize in Chemistry is awarded to J. B. Goodenough, M. S. Whittingham, and A. Yoshino for their outstanding contributions to the innovation and development of LIBs.^[^ ^4^ ^]^ At present, EVs and ESSs powered by LIBs are being promoted worldwide, which is of great significance for alleviating energy/environmental pressure and accelerating the great goal of carbon dioxide neutrality.

**Figure 1:**
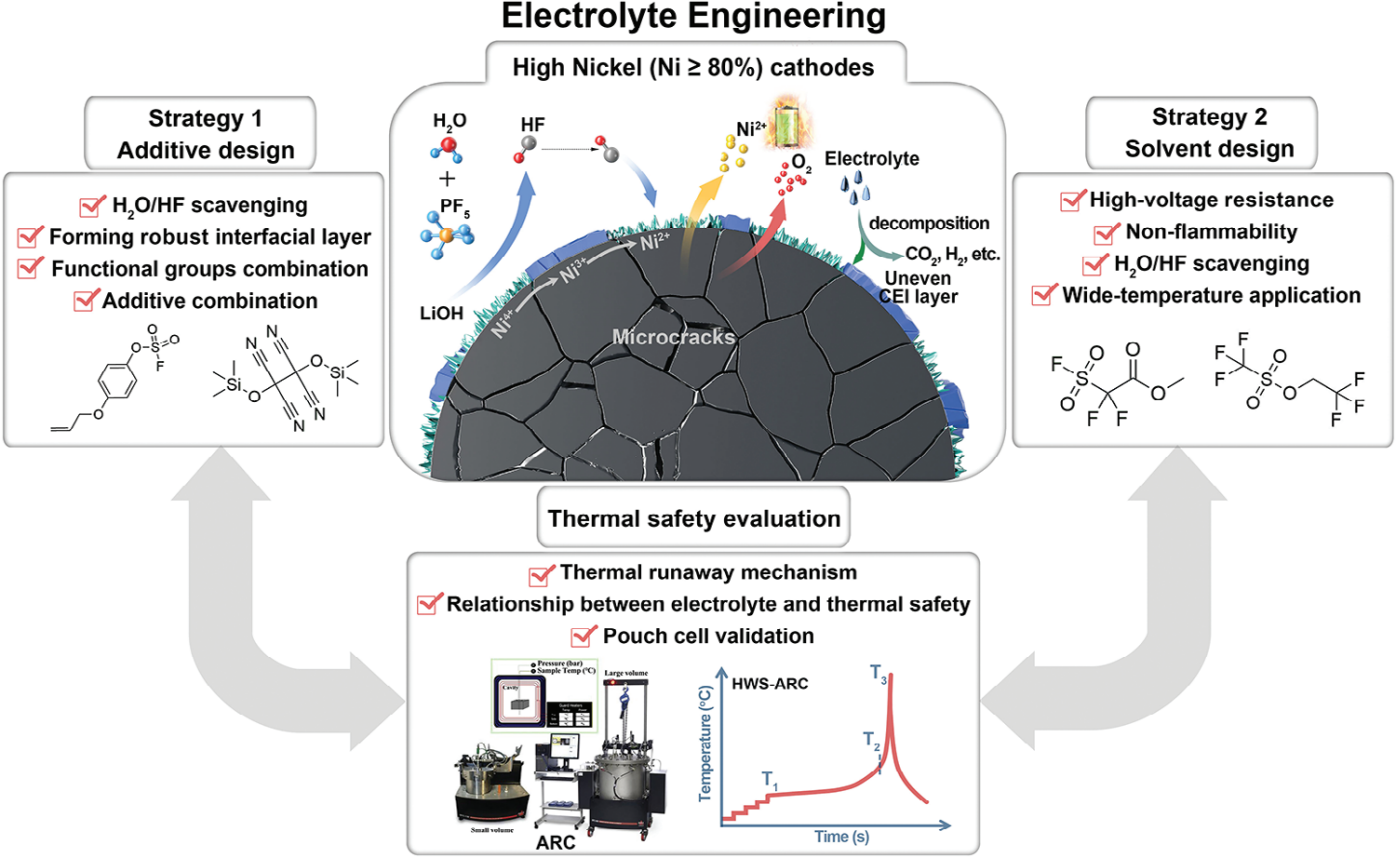
Schematic illustration of the pivotal challenges when using layered high‐Ni oxide cathode and the electrolyte engineering strategies for stabilizing layered high‐Ni oxide cathode.

In order to resolve the growing endurance anxieties, it is urgent to develop long‐life and high‐safety LIBs with higher energy density. In addition to adopting high‐capacity anode materials (e.g., Si, SiO_x_, and Li) to replace conventional graphite anode, the development of high‐voltage and high‐capacity cathode materials is also crucial in improving the energy density of LIBs.^[^ ^5^ ^]^ Wherein, high‐nickel (high‐Ni) oxide cathode materials (e.g., LiNi_x_Co_y_Mn_z_O_2_ (NCM_xyz_), x + y + z = 1, x ≥ 0.8) with layered crystal structure have aroused great interest due to their advantages like high theoretical specific capacity (180–250 mAh g^−1^), high operating voltage, and less usage of expensive Co, etc.^[^ ^6^, ^7^, ^8^, ^9^, ^10^, ^11^, ^12^, ^13^, ^14^, ^15^ ^]^ Pairing high‐Ni cathodes (Ni ≥ 80%) with the high specific capacity anode (such as SiO_x_–graphite or Si–graphite based composites) is one of the most important and widely accepted technical routes for developing next‐generation commercial LIBs with energy density exceeding 300 Wh kg^−1^. However, high‐Ni (Ni ≥ 80%) LIBs always suffer from poor cycle life and safety performance, hindering their large‐scale commercial applications. The main contradiction of layered high‐Ni oxide cathode is that the higher the Ni content, the higher the specific capacity, but the cycle stability and thermal stability become worse. Specifically, main problems and challenges of increasing Ni content in layered cathode materials are listed below (**Figure** **1**): 1) some residual lithium compounds (such as LiOH and Li_2_CO_3_) are inevitably formed during material synthesis and storage processes;^[^ ^16^, ^17^ ^]^ 2) the reduction of Ni^4+^ and HF formation (from reaction of LiOH with PF_5_, and trace water catalyzed electrolyte decomposition) can lead to damage of cathode/electrolyte interphase (CEI) layer and dissolution of transition metals (TMs) from high‐Ni cathode materials;^[^ ^1^, ^6^ ^]^ 3) with the increase of Ni content, lattice oxygen is more likely to be released and the thermal stability become worse;^[^ ^7^ ^]^ 4) mixed arrangement of Ni^2+^ (r = 0.069 nm) and Li^+^ (r = 0.076 nm) cations lead to transition of layered crystal structure to spinel and rock salt phases, hindering Li^+^ migration and leading to capacity loss;^[^ ^15^, ^18^ ^]^ 5) anisotropic lattice distortion and volume expansion/contraction during repeatedly delithiation and lithiation processes will cause inter‐particle cracking and intra‐particle cracking.^[^ ^15^ ^]^ Moreover, surface Li_2_CO_3_ residual, surface reconstruction (cation mixing and rock salt phase generation, etc.), Ni^4+^ reduction, etc. can lead to electrolyte oxidation to produce gaseous by‐products (e.g., O_2_, CO_2_).^[^ ^15^, ^17^, ^19^ ^]^ It is also noted that the migration of TMs and electrolyte oxidation species from cathode to anode will lead to the instability of the solid electrolyte interface phase (SEI) layer and accelerate capacity decay.^[^ ^9^, ^20^, ^21^, ^22^, ^23^, ^24^ ^]^

Resolving the above‐mentioned pivotal challenges of layered high‐Ni oxide cathode materials will greatly advance the commercialization of next‐generation long‐life high‐safety LIBs. Currently, to improve the performances of high‐Ni LIBs, great efforts have been devoted to electrolyte optimizations, ion‐doping, surface‐coating, and preparation methods improvement (e.g., concentration gradient design, single crystal material preparation, morphology control, etc.).^[^ ^6^, ^7^, ^8^, ^9^, ^10^, ^11^, ^12^, ^13^, ^14^, ^15^ ^]^ Among them, electrolyte optimization is one of the simplest and most powerful strategies to enhance the cycle life and safety of high‐Ni LIBs. Electrolyte usually consists of lithium salts, solvents, and functional additives (such as film‐forming additives, flame retardant additives, etc.).^[^ ^25^, ^26^, ^27^ ^]^ As an important component of LIBs, electrolytes not only serve as the carrier of Li^+^ transport between cathode and anode, but also regulate the electrode/electrolyte interphases.^[^ ^28^, ^29^, ^30^, ^31^, ^32^, ^33^, ^34^, ^35^, ^36^, ^37^, ^38^, ^39^ ^]^ Electrolytes should possess high Li^+^ mobility, excellent chemical/electrochemical/thermal stability, and a wide electrochemical window. In addition, comprehensive consideration of viscosity, melting point, toxicity, and inflammability is also very important, because slight changes in the physical or chemical properties of electrolytes may lead to significant changes in the performances of LIBs. Despite of their great success, conventional LiPF_6_‐carbonate‐based electrolytes with poor instability that are prone to generate unfavorable reactive species (such as HF, POF_3_, PF_5_, etc.) when exposed to trace of moisture, and operated at elevated temperatures and/or high voltages.^[^ ^40^, ^41^ ^]^ Another class of electrolytes is high voltage resistance and high safety novel electrolytes, which always adopt thermally stable lithium salts (e.g., lithium imides and lithium borates), solvents with high oxidative stability, and high flame‐retardant. In this review, we will comprehensively elaborate the recent progress of electrolyte engineering for next‐generation high‐Ni (Ni ≥ 80%) LIBs (full cells) with extremely aggressive chemistries, according to the classification of conventional LiPF_6_‐carbonate based electrolytes and high voltage resistance/high safety novel electrolytes. In addition, the critical roles of electrolytes in affecting the thermal safety of high‐Ni LIBs are also reviewed. This review will have an important impact on electrolyte innovation as well as the commercial evolution of high‐Ni (Ni ≥ 80%) LIBs, and also will be significant to break through the energy density ceiling of LIBs.

## 2 Design Principles of Functional Additives for LiPF_6_‐Carbonate‐Based Electrolytes of High‐Ni LIBs

**Figure 2:**
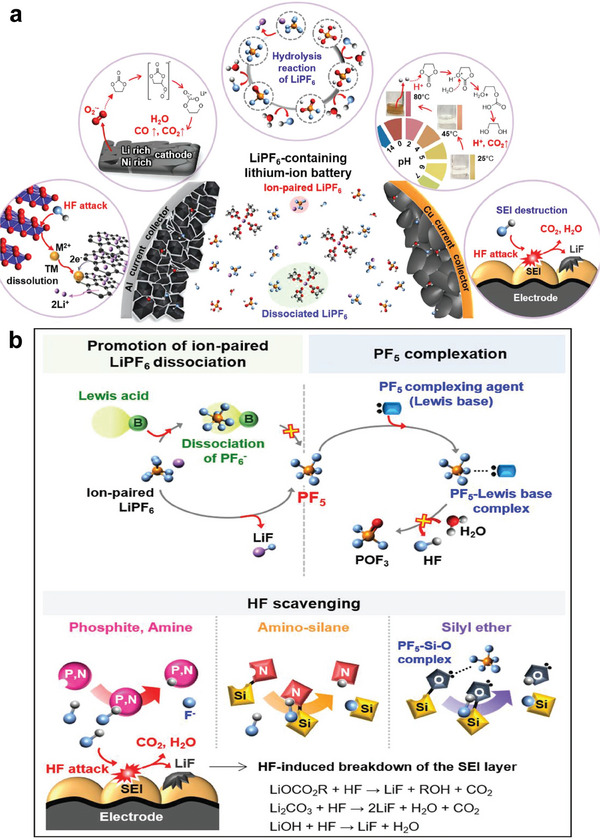
a) Schematic illustration of the pivotal challenges when using LiPF_6_‐carbonate‐based electrolytes. b) Schematic illustration of the strategies for stabilizing LiPF_6_‐carbonate‐based electrolytes. Reproduced with permission.^[^ ^40^ ^]^ Copyright 2019, Wiley‐VCH.

Conventional LiPF_6_‐carbonate‐based electrolytes are the most successful electrolytes for all types of LIBs. However, their stability will be greatly deteriorated when exposed to a trace of moisture, or used at elevated temperatures and/or high voltages.^[^ ^40^, ^41^ ^]^ The specific pivotal challenges when using LiPF_6_‐carbonate‐based electrolytes lie in the following aspects (**Figure** **2**a): 1) TMs dissolution caused by HF attack; 2) gas evolution from O_2_^•−^ triggered solvent decomposition; 3) LiPF_6_ hydrolysis to generate HF and other acids; 4) thermal induced decomposition of LiPF_6_‐based carbonate electrolytes; 5) destruction and reconstruction of CEI/SEI layers by HF attack. To stabilize LiPF_6_‐based carbonate electrolytes, the following directions are always considered (Figure 2b): 1) promoting the dissociation of ion‐paired

**Scheme 1:**
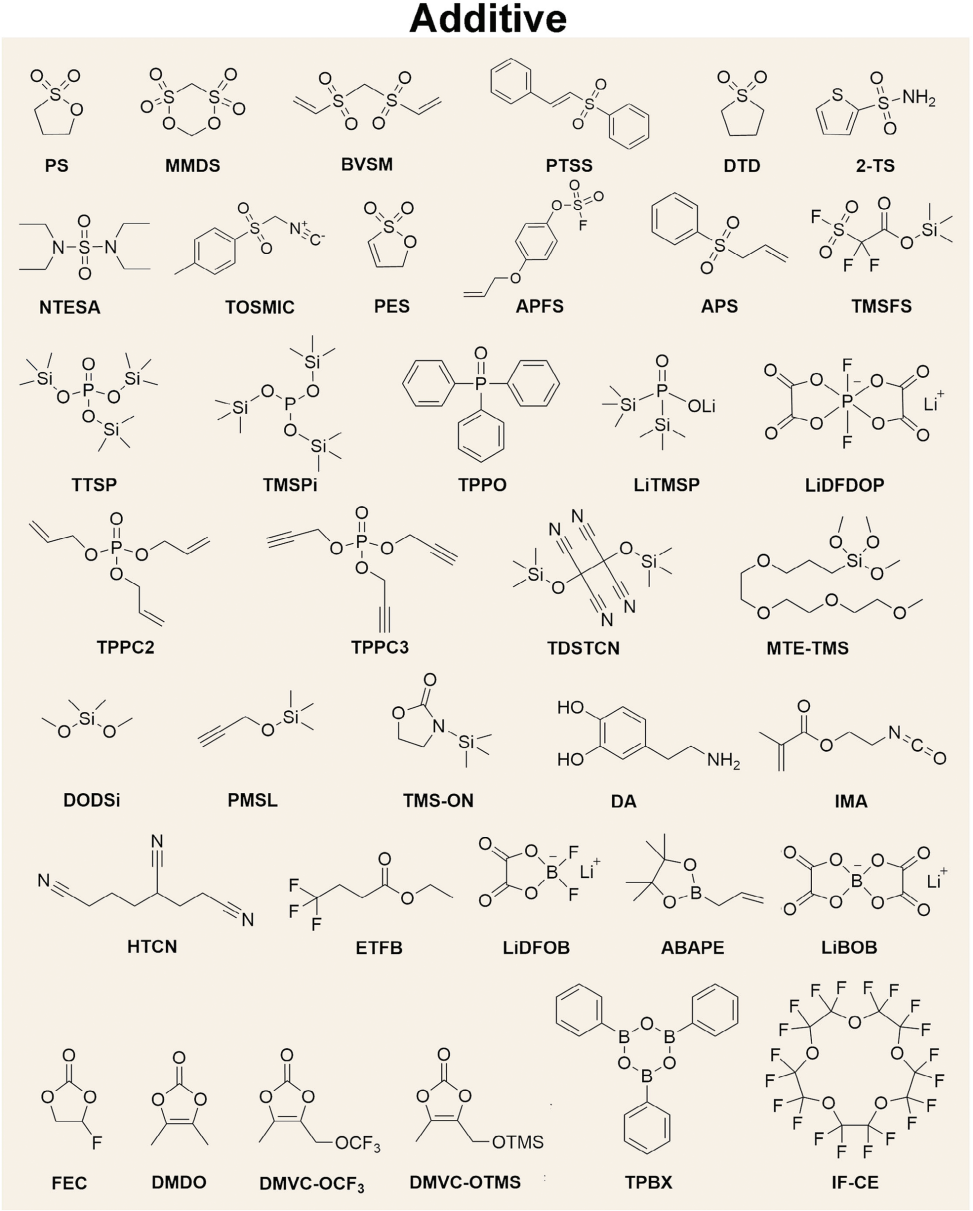
Chemical structure of additives for electrolytes of high‐Ni LIBs.

LiPF_6_, typically by electron‐deficient Lewis acids; 2) forming complex between PF_5_ and Lewis base (such as compounds with P, O, and N atoms bearing lone pairs) to hinder the hydrolysis of PF_5_; 3) scavenging HF and trace H_2_O by compounds containing electron‐donating sites such as nitrogen cores, silicon cores, amino silanes, and phosphite. Adding small amounts of functional additives (typically <5%) into conventional LiPF_6_‐carbonate‐based electrolytes is one of the most effective and economical strategies to enhance the cycle life and safety of LIBs.^[^ ^6^, ^9^, ^42^, ^43^, ^44^, ^45^, ^46^, ^47^, ^48^, ^49^, ^50^ ^]^ Ideal functional additives should not only possess the capability of scavenging detrimental species such as HF and H_2_O, but also exhibit self‐sacrifice in order to construct robust, stable, protective, and passivated CEI/SEI layers. Molecular orbital theory is usually used to guide the preliminary screening of film‐forming and self‐sacrificing additives: functional additives with a higher Highest Occupied Molecular Orbital than solvents will be preferentially oxidized to modify the CEI layer of cathode, while those with a lower Lowest Unoccupied Molecular Orbital than solvents will be preferentially reduced to modify the SEI layer of anode.^[^ ^42^, ^48^ ^]^ The action mechanisms of additives are greatly affected by the functional motif groups contained. In fact, previous studies have indicated that certain highly effective functional additives for low/medium‐Ni LIBs may not sufficiently improve the cycling performance of high‐Ni LIBs, as the battery chemistries become more aggressive with increasing Ni content beyond 80%.^[^ ^51^ ^]^ Currently, the functional additives capable of significantly enhancing the cycle life of high‐Ni (Ni ≥ 80%) LIBs are enumerated as follows (**Scheme** **1** and **Table** **1**): sulfur‐containing compounds (e.g., sulfonates and sulfates);^[^ ^52^, ^53^, ^54^, ^55^, ^56^, ^57^, ^58^, ^59^ ^]^ phosphorus‐containing compounds (e.g., organophosphates and phosphites);^[^ ^51^, ^52^, ^53^, ^60^, ^61^, ^62^, ^63^, ^64^, ^65^ ^]^ organosilicon compounds (e.g., siloxanes);^[^ ^50^, ^51^, ^53^, ^62^, ^65^, ^66^, ^67^, ^68^, ^69^, ^70^ ^]^ nitrogen‐containing compounds (e.g., amines, cyano and isocyano);^[^ ^50^, ^54^, ^55^, ^57^, ^69^, ^71^, ^72^, ^73^ ^]^ boron‐containing compounds (e.g., boron‐centered lithium salts);^[^ ^72^, ^74^, ^75^, ^76^, ^77^ ^]^ fluorinated organic compounds (e.g., fluorinated ethers and fluorinated carbonates);^[^ ^78^, ^79^, ^80^ ^]^ and carbonates (e.g., unsaturated carbonates and phenyl carbonates); etc.^[^ ^63^, ^65^, ^70^, ^81^, ^82^, ^83^ ^]^ Obviously, the approach of incorporating multiple functional motif groups into a single additive or adding several functional additives into an electrolyte is commonly employed to achieve synergistic effects for performance enhancement of high‐Ni (Ni ≥ 80%) LIBs.

**Table 1:** Representative electrolyte functional additives for high‐Ni LIBs.

Battery System	Electrolyte system	Performance	Ref.
Pouch‐type 4.4 Ah NCM811/Gr‐SiOx (2.5–4.2 V)	BE + 0.5 wt.% MMDS (BE = 1.0 m LiPF_6_ EC/EMC/DEC (3/5/2, by weight) containing 0.5 wt.% LiDFOB + 2 wt.% LiFSI + 2 wt.% FEC + 1 wt.% PS)	RT, 1 C rate, 560 cycles, capacity retention 79.84%	[53]
Coin‐type NCM811/graphite (2.8 –4.4 V) — mg cm^−2^	BE + 1 wt.% 2‐TS (BE = 1.0 M LiPF_6_ EC/EMC (1/2, by weight))	45°C,— rate, 115 cycles, capacity retention 68.68%	[54]
Coin‐type NCM811/graphite (— V) 9.50 ± 0.15 mg cm^−2^	BE + 0.25 wt.% NTESA (BE = 1.0 m LiPF_6_ EC/EMC (1/2, by volume))	RT, 1 C rate, 100 cycles, capacity retention 84.9%	[55]
Coin‐type NCM811/graphite (3–4.35 V) 3.78–4.51 mg cm^−2^	BE + 1 wt.% PTSS (BE = 1.0 m LiPF_6_ EC/DMC (3/7, by weight))	RT, 1 C rate, 100 cycles, capacity retention 63%	[56]
Coin‐type NCA/graphite (3–4.35 V)) — mg cm^−2^	BE + 3 wt.% TOSMIC (BE = 1.0 m LiPF_6_ EC/DEC (3/7, by weight))	25 °C, 0.5 C rate, 100 cycles, capacity retention 67%	[57]
Pouch‐type 1950 mAh NCM811/graphite (2.75–4.5 V))	BE + 1 wt.% DES (BE = 1.0 m LiPF_6_ EC/EMC (1/2))	25 °C, 2100 mA, 150 cycles, capacity retention 82.53%	[58]
Coin‐type NCM811/SiG‐C (2.5–4.2 V) 20.5 mg cm^−2^	BE + 0.5 wt.% APFS (BE = 1.0 m LiPF_6_ EC/EMC/DEC (25/45/30, by volume) containing 1 wt.% VC)	45 °C, 1 C rate, 300 cycles, capacity retention 72.5%	[59]
Coin‐type NCM90/SiO_x_‐C (2.7–4.3 V) 11 mg cm^−2^	BE + 0.5 wt.% APS (BE = 1.0 m LiPF_6_ EC/DEC (3/7, by weight)	30 °C, 0.5 C rate, 300 cycles, capacity retention 73.9%	[84]
Coin‐type NCM90/graphite (2.8–4.25 V) 2–2.5 mg cm^−2^	BE + 0.5 wt.% TMSFS (BE = 1.0 m LiPF_6_ EC/DEC (3/7, by weight)	30 °C, 0.5 C rate, 200 cycles, capacity retention ≈80%	[85]
Coin‐type NCM90/graphite (2.8–4.25 V) 3 mg cm^−2^	BE + 0.5 wt.% BVSM (BE = 1.0 m LiPF_6_ EC/DEC (3/7, by weight)	30 °C, 0.5 C rate, 200 cycles, capacity retention ≈80%	[86]
Pouch‐type 4.4 Ah NCM811/Gr‐SiOx (2.5–4.2 V)	BE + 0.5 wt.% TTSP (BE = 1.0 m LiPF_6_ EC/EMC/DEC (3/5/2, by weight) containing 0.5 wt.% LiDFOB + 2 wt.% LiFSI + 2 wt.% FEC + 1 wt.% PS)	RT, 1 C rate, 560 cycles, capacity retention 84.05%	[53]
Coin‐type NCM811/Gr‐Si (2.7 –4.4 V) ≈10 mg cm^−2^	BE + 2 wt.% TMSPi (BE = 1.0 m LiPF_6_ EC/DEC (1/1, by volume)	RT, 0.5 C rate, 50 cycles, capacity retention 80%	[51]
Coin‐type NCM811/graphite (2.75–4.2 V) 10.5–11.5 mg cm^−2^	BE + 1 wt.% TMSP‐i + 1 wt.% VC (BE = 1.0 m LiPF_6_ EC/DMC (1/1, by volume)	RT, C/3 rate, 200 cycles, capacity retention 91%	[65]
Coin‐type NCM811/graphite (2.8–4.3 V) 10.6 mg cm^−2^	BE + 0.5 wt.% TPPO (BE = 1.0 m LiPF_6_ EC/EMC (3/7, by weight)	RT, 0.5 C rate, 295 cycles, capacity retention 80%	[60]
Coin‐type NCM811/graphite (3–4.3 V) 2.6 mAh cm^−2^	BE + (0.4 ± 0.1) wt.% NaH_2_PO_4_ (BE = 1.0 m LiPF_6_ EC/EMC (3/7, by volume)	60 °C, 0.5 C rate, 150 cycles, capacity retention 75%	[61]
Coin‐type NCM811/graphite (3–4.25 V) 4.8 mg cm^−2^	BE + 1 wt.% LiTMSP (BE = 1.0 m LiPF_6_ EC/EMC (3/7, by volume)	Delivering better rate performance at −10 °C,	[62]
Pouch‐type 1668 mAh NCM811/graphite (3–4.2 V)	BE + 1 wt.% VC + 0.5 wt.% TPPC2 or TPPC3 (BE = 1.0 m LiPF_6_ EC/DEC (3/7, by weight))	55 °C, 1 C, 500 cycles, capacity retention 90.5%	[63]
Coin‐type NCM811/graphite (3–4.4 V) 9.6 mg cm^−2^	BE + 0.5 wt.% DODSi (BE = 1.0 m LiPF_6_ EC/EMC (1/2, by weight)	RT, — rate, 100 cycles, capacity retention 65.7%	[68]
Coin‐type NCM85/graphite (3–4.25 V) ≈12.3 mg cm^−2^	BE + 1 wt.% MTE‐TMS (BE = 1.0 m LiPF_6_ EC/DEC (3/7, by weight)	RT, C/5 rate, 100 cycles, capacity retention 84%	[88]
Coin‐type NCM811/Si‐C (2.5–4.3 V) 13.5 mg cm^−2^	BE + 0.5 wt.% VC + 0.5 wt.% DMVC‐OCF3 + 0.5 wt.% DMVC‐OTMS (BE = 1.15 m LiPF_6_ EC/EMC (3/7, by volume)	25 °C, 1 C rate, 400 cycles, capacity retention 81.5%	[70]
Pouch‐type 1000 mAh NCM811/graphite (3–4.3 V)	BE + 1 wt.% PMSL (BE = 1.0 m LiPF_6_ EC/DMC/EMC)	25 °C/60 °C, 0.5 C, 265 cycles, capacity retention ∼85%	[89]
Coin‐type NCM90/graphite (2.7–4.5 V) 5.5 ± 0.2 mg cm^−2^	BE + 0.5 wt.% TDSTCN (BE = 1.0 m LiPF_6_ EC/ EMC (3/7, by volume))	50 °C, 1 C, 200 cycles, capacity retention 83.2%	[50]
Coin‐type NCM90/graphite (2.7–4.25 V) 5.5 ± 0.2 mg cm^−2^	BE + 0.5 wt.% TDSTCN (BE = 1.0 m LiPF_6_ EC/ EMC (3/7, by volume))	RT, 1 C, 800 cycles, capacity retention 81.2%	[50]
Coin‐type LiNi_0.7_Co_0.15_Mn_0.15_O_2_/graphite (3–4.35 V) 24.9 mg cm^−2^	BE + 0.5 wt.% TMS‐ON (BE = 1.0 m LiPF_6_ EC/EMC/DEC (3/4/3, by volume) containing 1 wt.% VC)	45 °C, 0.5 C, 400 cycles, capacity retention 80.4%	[69]
Pouch‐type 1950 mAh NCM811/graphite (2.75–4.4 V)	BE + 0.5 wt.% IMA (BE = 1.0 m LiPF_6_ EC/EMC (1/2, by weight)	RT, 1 C, 150 cycles, capacity retention 81.9%	[73]
Coin‐type NCM83/graphite (3–4.5 V) — mg cm^−2^	BE + 3 wt.% HTCN (BE = 1.0 m LiPF_6_ EC/DEC (1/1, by volume)	55 °C, 1 C, 300 cycles, capacity retention 81.42%	[90]
LiNi_0.94_Co_0.06_O_2_/graphite (2.5–4.3 V) 4 mg cm^−2^	BE + 1.5 wt.% LiBOB (BE = 1.0 m LiPF_6_ EC/DEC (1/1, by volume)	RT, C/3, 500 cycles, capacity retention 80%	[75]
Coin‐type NCM83/graphite (2.8–4.3 V) 6 mg cm^−2^	BE + 1.5 wt.% LiDFOB (BE = 1.0 m LiPF_6_ EC/DMC/EMC (1/1/1, by volume)	RT, C/3, 200 cycles, capacity retention 83.1%	[77]
Pouch‐type NCA/graphite (2.75–4.2 V) — mg cm^−2^	BE + 1.5 wt.% ABAPE (BE = 1.2 m LiPF_6_ EC/EMC/DEC (3/5/2, by weight)	RT, 1 C, 500 cycles, capacity retention 87.7%	[76]
Coin‐type NCM811/graphite (3–4.25 V) 3.2 mg cm^−2^	BE + 5 wt.% TPBX (BE = 1 m LiPF_6_ EMC/EC/ DEC = 3/5/2, by weight)	RT, 0.5 C, 100 cycles, capacity retention 84%	[92]
Coin‐type NCM88/graphite‐SiO (2.5–4.35 V) 18.2 mg cm^−2^	BE + 0.4 wt.% IF‐CE + 2 wt.% FEC (BE unmentioned)	45 °C, 1 C, 100 cycles, capacity retention 88%	[78]
Coin‐type NCM70/graphite (3–4.35 V) 25.6 mg cm^−2^	BE + 1 wt.% ETFB (BE = 1.15 m LiPF_6_ EC/EMC/DEC (2/5/3, by weight)	45 °C, C/2, 300 cycles, capacity retention 84.8%	[79]
Pouch‐type 1000 mAh NCM811/graphite‐SiO_x_ (3–4.2 V)	BE + 30 wt.% FEC (BE = 1.0 m LiPF_6_ EC/DEC (3/7, by weight)	45 °C, 0.3 C, 100 cycles, capacity retention 83.3%	[80]

### 2.1 Sulfur‐Containing Compounds as Functional Additives for High‐Ni LIBs

**Figure 3:**
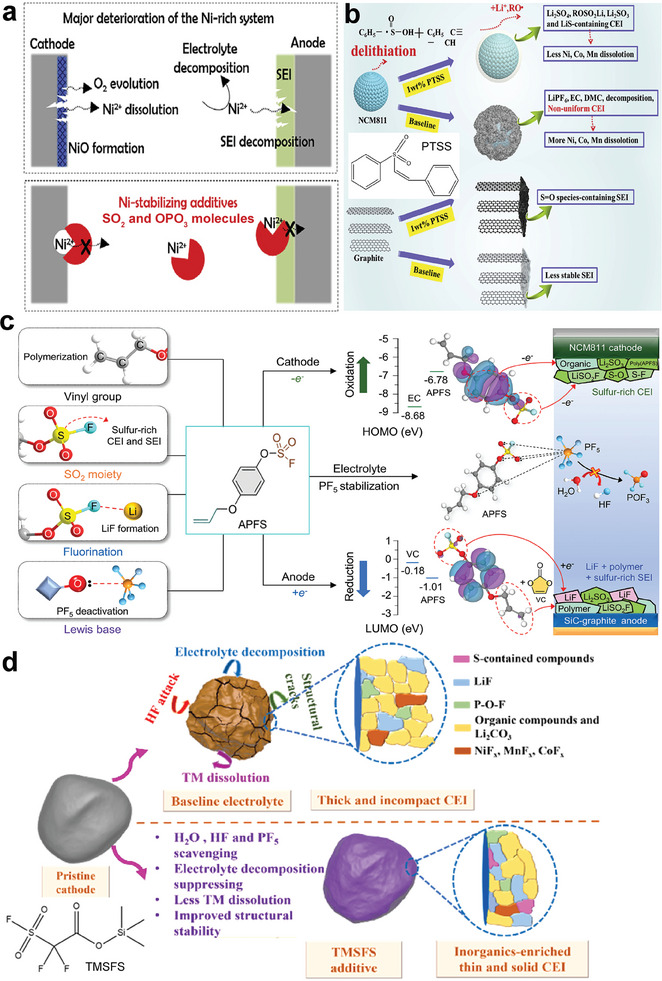
a) Schematic illustration of Ni‐stabilizing additives to eliminate the deterioration of high‐Ni LIBs. Reproduced with permission.^[^ ^52^ ^]^ Copyright 2019, Elsevier. b) Schematic illustration of the working mechanisms of PTSS in NCM811/graphite full cell. Reproduced with permission.^[^ ^56^ ^]^ Copyright 2020, Elsevier. c) Schematic illustration of the working mechanisms of APFS in NCM811/SiG‐C full cell. Reproduced with permission.^[^ ^59^ ^]^ Copyright 2023, Wiley‐VCH. d) Schematic illustration of the working mechanisms of TMSFS on NCM811cathode. Reproduced with permission.^[^ ^85^ ^]^ Copyright 2021, Elsevier.

It has been previously proposed that the development of Ni‐stabilizing electrolyte additives is a fundamental strategy to prevent the degradation of high‐Ni LIBs (**Figure** **3**a).^[^ ^52^ ^]^ The theoretically calculated Ni^2+^‐affinity serves as a key principle of screening the Ni‐stabilizing additives. In particular, SO_2_‐containing and OPO_3_‐containing molecules that are capable of strongly stabilizing Ni^2+^ in a structurally stable form show great potential as functional additives for high‐Ni LIBs. Conventional sulfonates and sulfates, such as 1,3‐propane sultone (PS), prop‐1‐ene‐1,3‐sultone (PES), methylene methanedisulfonate (MMDS) and 1,3,2‐dioxathiolane 2,2‐dioxide (DTD), can form interface layers enriched of ROSO_3_Li/ROSO_2_Li and Li_2_SO_4_/Li_2_SO_3_ components, which not only stabilize the interface layers, but also reduce the dissolution of metal ions from cathode and hinder the deposition of the dissolved metal ions at the anode.^[^ ^9^, ^42^, ^53^, ^54^ ^]^ For example, additives such as PES, MMDS, and DTD have improvement effect on the long cycle of NCM811/SiO_x_‐graphite.^[^ ^53^ ^]^ Considering the aggressive chemistries in high‐Ni LIBs, more functional groups or favorable elements are incorporated into S‐containing compounds. In virtue of thiophenes (self‐polymerizable at cathode to modify CEI layer) and N‐containing compounds (amines can scavenge H_2_O and HF), Zuo et al.^[^ ^54^ ^]^ investigate 2‐thiophene sulfonamide (2‐TS) as a multifunctional additive for performance enhancement of high voltage NCM811/graphite batteries at high temperatures. It is revealed that 2‐TS additive not only consumes the unfavorable H_2_O/HF species to alleviate the LiPF_6_ decomposition, but also preferentially reacts on the both NCM811 cathode and graphite anode to form uniform and dense low‐resistance interface layers. The concept of introducing amine functional groups into S‐containing compounds is also validated by N,N,N,N‐tetraethylsulfamide (NTESA) additive.^[^ ^55^ ^]^ Phenyl groups incorporated S‐containing compounds, such as phenyl trans‐styryl sulfone (PTSS)^[^ ^56^ ^]^ (Figure 3b), p‐toluenesulfonylmethyl isocyanide (TOSMIC),^[^ ^57^ ^]^ 4‐(allyloxy) phenyl fluorosulfate (APFS)^[^ ^59^ ^]^ and allyl phenyl sulfone (APS),^[^ ^84^ ^]^ are also highly effective additives for high‐Ni LIBs. The most representative one is APFS^[^ ^59^ ^]^ (Figure 3c), which contains functional groups of sulfate (forming sulfur‐rich CEI and SEI layer), phenyl (benefiting for oxidative polymerization, poly(APFS)), unsaturated vinyl group (copolymerizing with vinylene carbonate (VC) at anode), Lewis base oxygen (deactivating the Lewis acid PF_5_ and suppressing HF formation) and monofluorine (contributing to LiF formation at anode). As a result, the combination of APFS and VC enables NCM811/SiG‐C full cell with a high‐capacity retention of 72.5% after 300 cycles at 45 °C. Moreover, the importance of incorporating siloxane groups (trimethylsilyl 2‐(fluorosulfonyl) difluoroacetate (TMSFS),^[^ ^85^ ^]^ Figure 3d), unsaturated groups (APFS,^[^ ^59^ ^]^ APS,^[^ ^84^ ^]^ bis(vinylsulphonyl) methane (BVSM)^[^ ^86^ ^]^) and cyano‐groups (TOSMIC)^[^ ^57^ ^]^ are also revealed.

### 2.2 Phosphorus‐Containing Compounds as Functional Additives for High‐Ni LIBs

**Figure 4:**
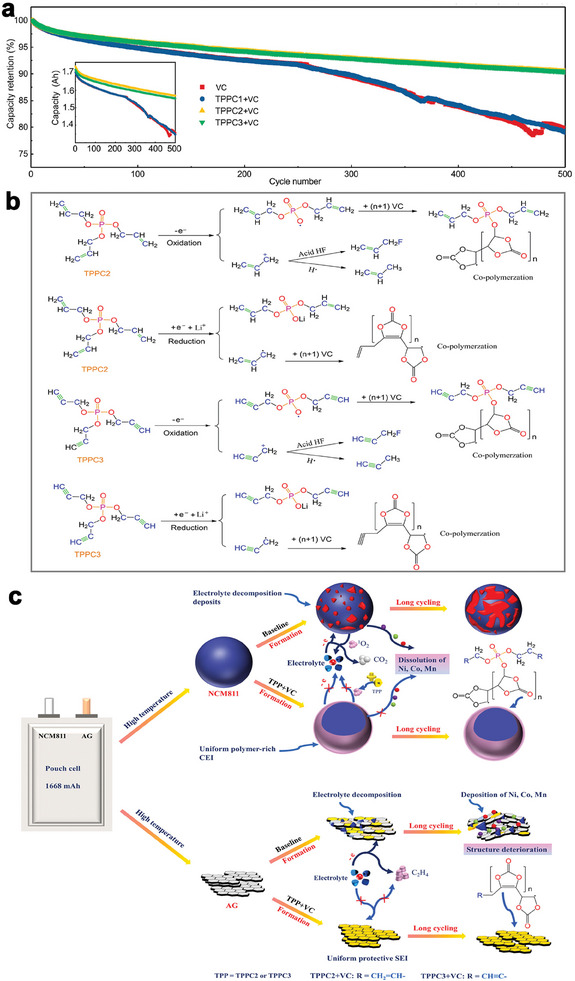
a) Cycling performance of NCM811/AG pouch cells at 1 C and 55C with different electrolyte systems (TPPC1 is tripropyl phosphate, TPPC2 is triallyl phosphate and TPPC3 is tripropargyl phosphate). b) Schematic illustration of the construction of protective interface layers on NCM811cathode and AG anode. c) Proposed working mechanisms for the TPPC2 and TPPC3 with VC via oxidation and reduction reactions. Reproduced with permission.^[^ ^63^ ^]^ Copyright 2021, Elsevier.

As shown in Figure 3a, OPO_3_‐containing molecules can also serve as Ni‐stabilizing additives for high‐Ni LIBs.^[^ ^52^ ^]^ It is interestingly shown that tris(trimethylsilyl) phosphate (TTSP) additive enables NCM811/SiO_x_‐graphite with superior cycling stability compared to sulfur‐containing additives such as PES, MMDS, and DTD.^[^ ^53^ ^]^ Another typical phosphorus‐containing additive is tris(trimethylsilyl) phosphite (TMSPi), which can participate in the formation of stable and robust CEI and SEI layers, scavenge HF, inhibit “crosstalk” of CF_x_ species from cathode to anode.^[^ ^51^, ^65^, ^66^, ^67^ ^]^ The O─Si bond cleavage pathway produces a series of PO_3_‐containing species, such as P(OSi(CH_3_)_3_)_2_OH, P(OSi(CH_3_)_3_) (OH)_2_, and P(OH)_3_. These PO_3_‐containing species will be oxidized earlier than carbonates to participate in the formation of compact CEI layer. Moreover, O─Si bond cleavage is a favorable pathway to scavenge HF.^[^ ^66^ ^]^ Besides, the products of the O─Si bond cleavage from TMSPi can participate in the formation of SEI layer on graphite anodes due to their reactivity with functional groups formed by carbonates degradation.^[^ ^67^ ^]^ Then, a series of other novel phosphorus‐containing compounds, such as triphenylphosphine oxide (TPPO, increasing initial Coulombic efficiency),^[^ ^60^ ^]^ monobasic sodium phosphate (NaH_2_PO_4_, enhancing cycling stability at 60 °C),^[^ ^61^ ^]^ lithium bis(trimethylsilyl) phosphate (LiTMSP, improving rate performance at −10 °C),^[^ ^62^ ^]^ unsaturated phosphates,^[^ ^63^ ^]^ and lithium difluoro(dioxalato)phosphate (LiDFDOP, validating in single crystal NCM811/ artificial graphite (AG) pouch cell at room and high temperatures),^[^ ^64^ ^]^ are preliminarily investigated as effective additive for cycling performance enhancement of high‐Ni LIBs. Wherein, when combined with VC additive, unsaturated phosphates (triallyl phosphate (TPPC2) and tripropargyl phosphate (TPPC3)) endow NCM811/AG pouch cells with high‐capacity retention of 90.5% and 90.4% after 500 cycles at 1 and 55 °C, respectively (**Figure** **4**a).^[^ ^63^ ^]^ The additive decomposition for SEI and CEI layers building is presented in Figure 4b. The TPPC2 and TPPC3 oxidatively and reductively decompose on both cathode and anode to produce various radicals, and then these radicals can react with the double bonded moiety of the VC to form robust cross‐linked polymer SEI and CEI layers. Meanwhile, the radicals produced through oxidative decomposition can further combine with the harmful species HF, mitigating the corrosion of F‐containing species to cathode surface. Under the help of dual‐additive electrolyte, less Li_2_CO_3_, LiF, LiPF_x_O_y_ and transition metal species are contained in the SEI layer while the CEI layer are enriched of thermally stable polymer species (Figure 4c).^[^ ^63^ ^]^ The robust and homogenous SEI/CEI layers greatly alleviate the electrolyte decompositions, transition metal dissolution/deposition and active oxygen releasing.

### 2.3 Organosilicon Compounds as Functional Additives for High‐Ni LIBs

**Figure 5:**
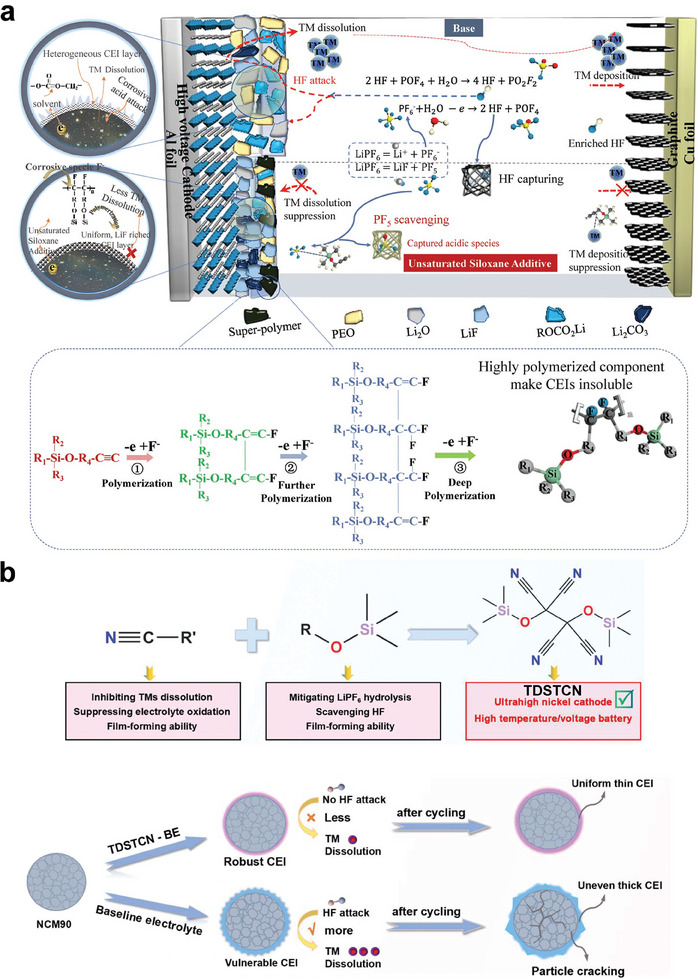
a) Origins of multifunctionalities of unsaturated siloxane additive. The merits of stabilizing both interphase and electrolyte, and the possible electrochemical polymerization reaction routine with a high unsaturation bond. Reproduced with permission.^[^ ^89^ ^]^ Copyright 2022, Wiley‐VCH. b) Schematic illustration of the working mechanism of TDSTCN additive on the NCM90 cathode. The NCM90/Gr full cells were cycled at 50 °C for 100 cycles. Reproduced with permission.^[^ ^50^ ^]^ Copyright 2023, Wiley‐VCH.

The most representative organosilicon additives are siloxanes, which contains Si─O bonds that can react with HF to form stable Si─F bonds.^[^ ^50^, ^51^, ^53^, ^62^, ^65^, ^66^, ^67^, ^68^, ^69^, ^70^, ^85^, ^87^, ^88^, ^89^ ^]^ In the above examples, the combinations of Si─O bonds with sulfur (TMSFS^[^ ^85^ ^]^) or phosphorus (TMSPi/TTSP,^[^ ^51^, ^53^, ^65^, ^87^ ^]^ LiTMSP^[^ ^62^ ^]^) in one additive have already been proved to be effective in high‐Ni LIBs. Other evaluated siloxanes includes dimethoxydimethylsilane (DODSi),^[^ ^68^ ^]^ methoxytriethyleneoxypropyltrimethoxysilane (MTE‐TMS),^[^ ^88^ ^]^ unsaturated siloxanes,^[^ ^70^, ^89^ ^]^ cyano‐siloxane (TDSTCN),^[^ ^50^ ^]^ etc. It is reported that siloxanes with unsaturated bonds (e.g., C═C and C≡C) will electrochemically polymerized to form a robust insoluble CEI layer enriched of [Si─O─R]_n_ polymer species and inorganic LiF (**Figure** **5**a),^[^ ^89^ ^]^ suppressing the cathode–electrolyte parasitic reactions, TMs dissolving, gas evolution, and impedance growth. As a result, only 1 wt. % unsaturated siloxane additive (propargyloxytrimethylsilane (PMSL)) enables 4.3 V NCM811/graphite pouch cell with a stable cycling at 60 °C, delivering a high capacity retention of 85% over 265 cycles. Very recently, cyano‐groups are innovatively incorporated into a siloxane to synthesize TDSTCN (Figure 5b),^[^ ^50^ ^]^ which enables ultrahigh nickel LiNi_0.9_Co_0.05_Mn_0.05_O_2_/graphite (NCM90/Gr) full cells with dramatically increased cycle life, especially at 50 °C and high voltage (4.5 V). It is revealed that TDSTCN additive can inhibit TMs dissolution from NCM90 cathode by forming a robust CEI layer enriched with cyano‐groups, which can form strong complex with TMs. In advantage of Si─O bonds, TDSTCN additive can scavenge HF and prevent formation of erosive HF from LiPF_6_ hydrolysis. Moreover, TDSTCN additive can suppress thermal‐induced graphite anode exfoliation by forming a SEI layer enriched with LiF. Except siloxanes, another organosilicon additives are aminosilanes (N─Si, such as 3‐(trimethylsilyl)−2‐oxazolidinone (TMS‐ON)^[^ ^69^ ^]^), which are also suitable for scavenging both HF and H_2_O.

### 2.4 Other Compounds as Functional Additives for High‐Ni LIBs

There are mainly two classes of nitrogen‐containing compounds as additives: amines and cyano/isocyano‐containing compounds, both of which are Lewis base and have the ability to suppress PF_5_ hydrolysis and scavenge HF/H_2_O.^[^ ^1^, ^40^ ^]^ In the above examples, the combinations of nitrogen with sulfur (2‐TS,^[^ ^54^ ^]^ NTESA^[^ ^55^ ^]^), phenyl (TOSMIC,^[^ ^57^ ^]^ dopamine (DA)^[^ ^72^ ^]^) or silicon (TDSTCN,^[^ ^50^ ^]^ TMS‐ON^[^ ^69^ ^]^) in one additive have already been proved to be effective in high‐Ni LIBs. Serving as additives, cyano/isocyano‐containing compounds, such as TDSTCN,^[^ ^50^ ^]^ TOSMIC,^[^ ^57^ ^]^ isocyanoethyl methacrylate (IMA),^[^ ^73^ ^]^ 1,3,6‐hexanetricarbonitrile (HTCN),^[^ ^90^ ^]^ can form a strong complex with TMs, effectively preventing the TMs dissolution and improving the thermal stability of cathode.^[^ ^91^ ^]^

**Figure 6:**
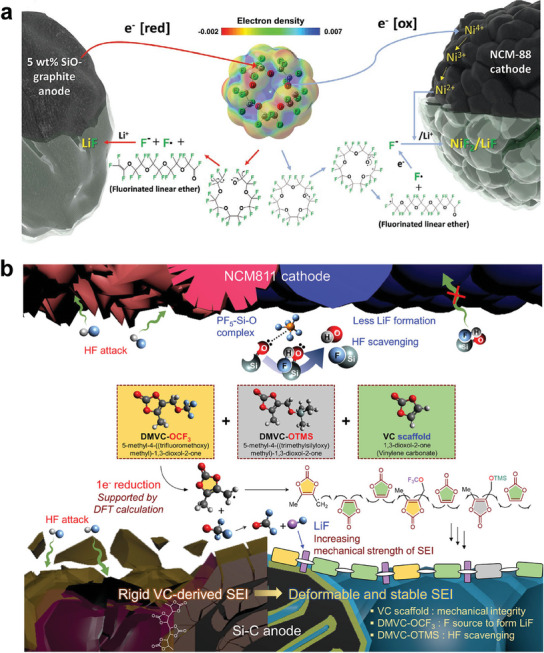
a) Proposed working mechanism of fluorinated ether additive (IF‐CE) (induce the formation of perfluoro ether, LiF, and Li_x_PF_y_ species). Reproduced with permission.^[^ ^78^ ^]^ Copyright 2023, Wiley‐VCH. b) Unique features of DMVC‐OCF3, DMVC‐OTMS, and VC for building stable interfacial layers. Reproduced with permission.^[^ ^70^ ^]^ Copyright 2021, Springer Nature.

Boron‐containing additives not only facilitate the formation of a robust stable interface layer enriched with Li_x_B_y_O_z_ and B_x_O_y_ species^[^ ^72^, ^74^, ^75^, ^76^, ^77^, ^92^ ^]^ but also promote the dissociation of ion‐paired LiPF_6_.^[^ ^40^ ^]^ Lithium borates (such as lithium bis(oxalate) borate (LiBOB)^[^ ^72^, ^74^, ^75^ ^]^ and lithium difluoro(oxalato)borate (LiDFOB)^[^ ^77^ ^]^) have already been proved to be effective in high‐Ni LIBs. For example, it is reported that the capacity retention of ultrahigh‐Ni LIBs (LiNi_0.94_Co_0.06_O_2_/graphite) after 500 cycles is increased from 61% to 80% by LiBOB additive.^[^ ^75^ ^]^ In other cases, incorporating the unsaturated vinyl group (allylboronic acid pinacol ester (ABAPE)^[^ ^76^ ^]^) and phenyl group (2,4,6‐triphenyl boroxine (TPBX)^[^ ^92^ ^]^) into borates will induce the formation of robust stable polymeric‐type CEI layer on high‐Ni cathode surface, alleviating electrolyte oxidation and TMs dissolution. The most dominant advantage of fluorinated additives, such as APFS,^[^ ^59^ ^]^ TMSFS,^[^ ^85^ ^]^ LiDFDOP,^[^ ^64^ ^]^ LiDFOB,^[^ ^77^ ^]^ icosafluoro‐15‐crown‐5‐ether (IF‐CE, **Figure** **6**a),^[^ ^78^ ^]^ ethyl 4,4,4‐trifluorobutyrate (ETFB),^[^ ^79^ ^]^ fluoroethylene carbonate (FEC),^[^ ^78^, ^80^ ^]^ is forming stable and robust CEI/SEI layers enriched of LiF species. And these fluorinated additives are more effective in LIBs consisting of high‐Ni cathode and silicon‐graphite composite anode.

VC is the most widely used functional additive in varied conventional LIB systems. However, VC is more effective in high‐Ni LIBs only when used together with other functional additives, such as VC+ TPPC^[^ ^63^ ^]^ and VC+ DTD.^[^ ^83^ ^]^ Then, some VC derivatives, such as 4,5‐Dimethyl‐1,3‐dioxol‐2‐one (DMDO),^[^ ^82^ ^]^ 5‐methyl‐4‐((trifluoromethoxy)methyl)−1,3‐dioxol‐2‐one (DMVC‐OCF3)^[^ ^70^ ^]^ and 5‐methyl‐4‐((trimethylsilyloxy)methyl)−1,3‐dioxol‐2‐one (DMVC‐OTMS),^[^ ^70^ ^]^ have been investigated to enhance the cycle life of high‐Ni LIBs. For example, the copolymerization of VC with DMVC‐OCF3 and DMVC‐OTMS benefits the formation of a deformable and stable SEI on Si–C anode (Figure 6b).^[^ ^70^ ^]^ Moreover, DMVC‐OCF3 provides an F source to form LiF and DMVC‐OTMS scavenges erosive HF. As a result, these VC‐type additives enable single crystal NCM811/Si‐C LIBs with 81.5% capacity retention after 400 cycles at 1 C and fast charging capability.

In summary of this part, as for the development of functional additives for conventional LiPF_6_‐carbonate‐based electrolytes, not only the H_2_O/HF inhibiting and scavenging ability but also the forming ability of robust stable interface layer are required to be considered. Obviously, the synergistic effects achieved by incorporating multiple functional motif groups into a single additive or adding several functional additives into an electrolyte are very important to significantly enhance the cycling performances of high‐Ni (Ni ≥ 80%) LIBs. However, there are still several issues to be resolved:

1) The working mechanisms of different functional additives or different functional motif groups (Scheme 1) are still not comprehensive. In the future, their detailed working mechanisms must be clarified based on the detailed characterization of varied interface byproducts (such as solid‐state byproducts, gas byproducts, and soluble byproducts, etc.). Also, it is necessary to depict the decomposition pathways of the functional additive itself on both high‐Ni cathode and anode. In addition, the effects of functional additives or functional motif groups on the electrolyte oxidative/reductive pathways require clarification. Moreover, the effects of functional additives on the thermal stability of electrodes and the thermal safety of large‐format high‐Ni LIBs should be evaluated.
2) The structure screening of functional additives is still mainly relies on expensive and low‐efficient trial‐and‐error experiments. In the future, based on the understanding of the aggressive chemistries in high‐Ni LIBs and the working mechanisms of confirmed functional additives, it is necessary to adopt high throughput computing and experimenting methods to design and synthesize a series of potentially high effective functional additives containing different functional motif groups. Also, the synthesis methods of functional additives are required to be feasible, low‐cost, and environmentally friendly. It is also noted here that the design, synthesis, and evaluation of novel functional additives require the cooperation of researchers who are experienced and knowledgeable in the different fields of theoretical chemistry, organic synthesis, and battery research.
3) It is noted here (Table 1) that the cycling stability of coin‐type high‐Ni LIBs is always poor, while the pouch‐type high‐Ni LIBs exhibit much superior cycle life under the same conditions (e.g., the same LiPF_6_‐carboante electrolyte and areal capacity). Additionally, it can be noted that there is a significant difference in the areal capacity of high‐Ni cathodes (from 4.6 to 22 mg cm^−2^) used in different reports. It is hard to demonstrate the reliability of functional additives in highly aggressive chemistry conditions just using LIBs with low‐loading cathodes (<6 mg cm^−2^). Therefore, the effectiveness of functional additives must be evaluated in both coin‐type and pouch‐type high‐load (>15 mg cm^−2^) high‐Ni full cells.

## 3 Design Principles of High Voltage Resistance/High Safety Electrolytes for High‐Ni LIBs

**Figure 7:**
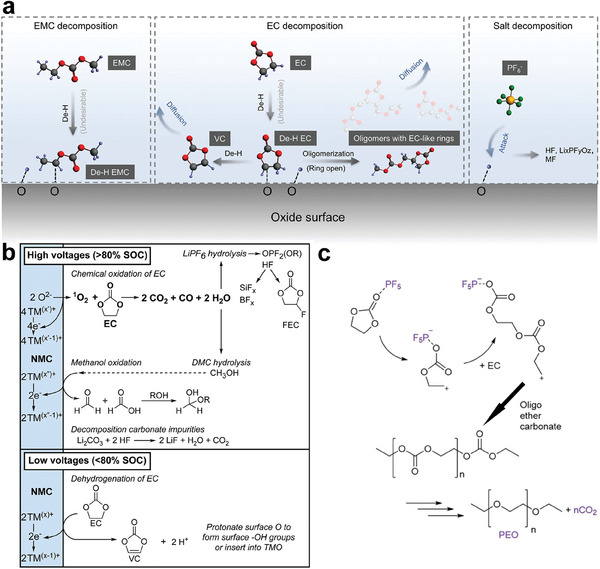
a) Proposed electrolyte oxidation pathways via carbonate dehydrogenation. Reproduced with permission.^[^ ^93^ ^]^ Copyright 2020, Royal Society of Chemistry. b) Depiction of the electrolyte decomposition pathways that occur at high‐Ni cathode at high potentials and low potentials. Reproduced with permission.^[^ ^94^ ^]^ Copyright 2022, Royal Society of Chemistry. c) PF_5_‐catalyzed ring‐opening polymerization of EC. Reproduced with permission.^[^ ^40^ ^]^ Copyright 2019, Wiley‐VCH.

**Scheme 2:**
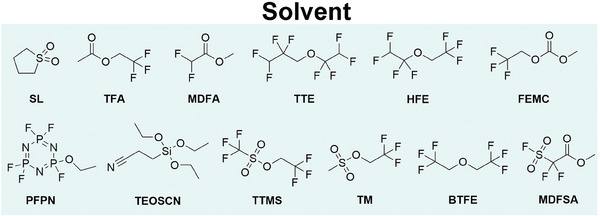
Chemical structure of solvents for electrolytes of high‐Ni LIBs.

Conventional LiPF_6_‐carbonate‐based electrolytes are normally prepared by dissolving LiPF_6_ into the mixed solvents of high dielectric constant cyclic carbonates (such as ethylene carbonate (EC), propylene carbonate (PC)) and low viscosity linear alkyl carbonates (such as dimethyl carbonate (DMC), ethyl methyl carbonate (EMC), and diethyl carbonate (DEC)). Wherein, EC solvent is conventionally regarded as the indispensable component favoring the formation of high‐quality SEI layer on a graphite‐based anode.^[^ ^25^, ^26^ ^]^ However, in recent years, EC is confirmed as the least oxidation‐resistant carbonate solvent, limiting the high voltage (>4.3 V, vs Li^+^/Li) application of conventional LiPF_6_‐carbonate‐based electrolytes.^[^ ^23^, ^26^, ^40^, ^93^, ^94^, ^95^ ^]^ Several mechanisms, such as the dehydrogenation reaction mechanism (**Figure** **7**a,b)^[^ ^93^, ^94^ ^]^ and ring‐opening reaction mechanism (Figure 7c)^[^ ^40^ ^]^ have been proposed to explain oxidative decompositions of EC solvent. The cross‐talking effects of proton‐containing side products and TMs are also severe in high‐Ni LIBs when using conventional LiPF_6_‐carbonate‐based electrolytes.^[^ ^23^, ^24^, ^96^ ^]^ In addition, conventional LiPF_6_‐carbonate‐based electrolytes are thermally/moisture unstable (ascribing to LiPF_6_) and highly flammable. Therefore, it is necessary to develop high‐voltage resistance and high safety electrolytes for high‐Ni LIBs. The commonly used strategies to develop novel electrolytes^[^ ^26^ ^]^ are adopting thermally highly stable lithium salts (such as LiBOB, LiDFOB, lithium bis(trifluoromethanesulfonyl)imide (LiTFSI), lithium bis(fluorosulfonyl)imide (LiFSI)), solvents with high oxidative stability (such as fluorinated ethers, fluorinated carbonates, fluorinated carboxylic acid esters, sulfone, fluorinated sulfones, nitrile, etc.) and high flame‐retardant (such as phosphate esters, fluorocyclophosphazenes, fluorinated solvents, etc.) (**Scheme** **2**), while decreasing the incorporation of LiPF_6_ and carbonate solvents. In the following, we will elaborate the reported novel electrolytes for high‐performance high‐Ni (Ni ≥ 80%) LIBs, according to two classifications: Medium/low concentration (≤3 m) electrolytes; High‐concentration electrolytes (HCEs, ≥ 3 m) and localized high‐concentration electrolytes (LHCEs).

### 3.1 Medium/Low Concentration Electrolytes for High‐Ni LIBs

**Figure 8:**
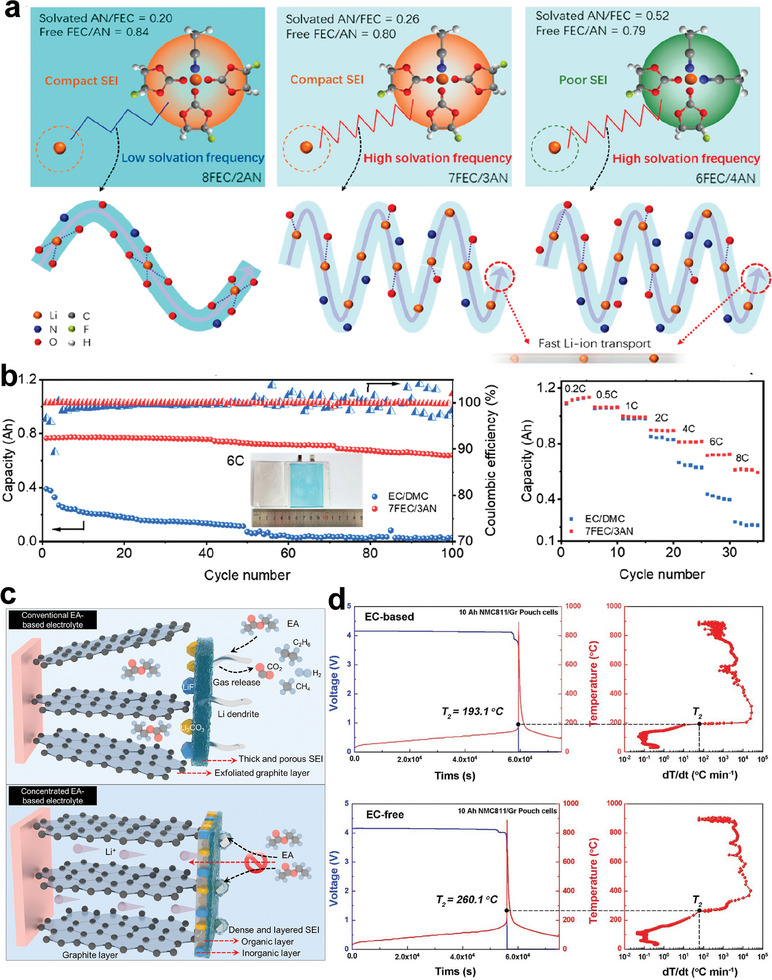
a,b) The designed 1 m LiPF_6_ 7FEC/3AN electrolyte possesses the film‐forming solvation sheaths and ion‐hopping‐assisting channels simultaneously, enabling a compact SEI layer for high‐rate cycling of NCM811/graphite pouch cells. Reproduced with permission.^[^ ^99^ ^]^ Copyright 2022, American Chemical Society. c) In the 3.0 m LiPF_6_ EA/FEC, the dense and uniform SEI is formed by the joint participation of rich anions and FEC, preventing side reactions between EA and plated Li, thus maintaining a stable graphite–electrolyte interface during long‐term low‐temperature cycling. Reproduced with permission.^[^ ^101^ ^]^ Copyright 2023, Elsevier Inc. d) ARC‐tested safety features of 10 Ah NCM811/graphite pouch cells with the ethylene carbonate (EC) based electrolyte and EC‐free electrolyte. Reproduced with permission.^[^ ^108^ ^]^ Copyright 2021, Wiley‐VCH.

The first class of novel electrolytes is formulated by LiPF_6_ and high voltage stable solvents with different functional groups (Scheme 2 and **Table** **2**), such as sulfolane (SL),^[^ ^97^ ^]^ FEC,^[^ ^97^, ^99^, ^100^, ^101^, ^102^, ^103^, ^104^, ^105^, ^106^, ^107^ ^]^ 2,2,2‐trifluoroethyl acetate (TFA),^[^ ^98^, ^106^ ^]^ acetonitrile (AN),^[^ ^99^, ^100^ ^]^ methyl difluoroacetate (MDFA),^[^ ^102^ ^]^ ethoxy‐pentafluoro‐cyclotriphosphazene (PFPN),^[^ ^102^ ^]^ 2,2,2‐trifluoroethyl methyl carbonate (FEMC),^[^ ^103^, ^104^, ^105^ ^]^ 1,1,2,2‐tetrafluoroethyl‐2′,2′,2′‐trifluoroethyl ether (HFE).^[^ ^103^, ^104^ ^]^ Solvents with different functional groups endow novel electrolytes with various characteristics. For example, the TFA solvent has a highly fluorinated CF_3_ functional group with an electron‐withdrawing effect, endowing high thermodynamic and electrochemical stability to electrolytes. Thus, the flame resistance electrolyte of 1 m LiPF_6_ PC/TFA (3/7 by volume) enables 730 mAh NCM811/graphite pouch cells with long cycle life (82% capacity retention at 1 C and 45 °C for 400 cycles) and high safety (pass overcharge testing).^[^ ^98^ ^]^ Additionally, a judicious choice of solvent can effectively improve the rate capability and low‐temperature performance of high‐Ni LIBs.^[^ ^99^, ^100^, ^101^, ^102^ ^]^ Fan et al. design a fast‐charging highly conductive electrolyte (1 m LiPF_6_‐FEC/AN, 7/3 by volume), enabling extreme fast charging (XFC) of graphite anode (exceeding 20 C) and high voltage high‐rate operation (exceeding 4.4 V) of NCM811/graphite pouch cells (**Figure** **8**a‐b).^[^ ^99^, ^100^ ^]^ The XFC performance of batteries in the LiPF_6_‐FEC‐AN electrolyte is derived from the low desolvation energy of Li^+^, Li^+^ hopping‐assisting nature of AN, and the formation of low‐resistance SEI layers.^[^ ^99^, ^100^ ^]^ Q. Zhang et al.^[^ ^101^ ^]^ and J. Ming et al.^[^ ^102^ ^]^ using (fluorinated) linear carboxylate esters with low freezing point and low viscosity as co‐solvents to formulate 3.0 m LiPF_6_ ethyl acetate (EA)/FEC (9:1 by volume, Figure 8c) and LiPF_6_/MDFA/PFPN/FEC (1:7:0.5:1 by molar), respectively, to enhance the performances of high‐Ni LIBs at subzero temperatures. As for safety issues, perfluorinated electrolytes have higher thermal stability and can promote the formation of uniform protective CEI layers with high inorganic content. For example, accelerated rate calorimeter (ARC) testing demonstrates that NCM811/graphite pouch cells adopting a novel perfluorinated electrolyte (1 m LiPF_6_ 0.02 m LiDFOB in FEC:HFE:FEMC, 2:2:6 by volume) deliver higher thermal runaway onset temperature (*T*_2_).^[^ ^103^, ^104^ ^]^ Recently, some researchers have pointed out that EC is prone to occur oxidative decomposition when used with high‐nickel layered oxide cathodes (such as NCM811), rendering it unsuitable for next‐generation LIBs.^[^ ^107^, ^108^, ^109^ ^]^ It is proposed that developing EC‐free electrolytes is very important to enhance the high‐voltage cycle life and safety of high‐Ni LIBs.^[^ ^107^, ^108^, ^109^ ^]^ For example, ARC results suggest (Figure 8d) that a designed EC‐free electrolyte of 0.8 m LiFSI‐0.1 m LiTFSI‐0.6 m LiPF_6_ EMC will increase *T*_2_ of 10 Ah NCM811/graphite pouch cells by 67 °C compared to conventional EC‐based electrolyte (1.0 m LiPF_6_ EC‐EMC (3:7)).^[^ ^108^ ^]^

**Table 2:** Representative high voltage resistance and high safety electrolytes for high‐Ni LIBs.

Battery System	Electrolyte System	Performance	Ref.
Coin‐type NCM811/Gr‐SiOx (2.75–4.5 V) 22 mg cm^−2^	1 m LiPF_6_ DMC/SL/FEC/HFE (30/30/20/20 by weight)	RT, C/2 rate, 200 cycles, capacity retention 77.51%	[97]
Pouch‐type 730 mAh NCM811/graphite (2.7–4.3 V) 12.1 mg cm^−2^	1 m LiPF_6_ PC/TFA (3/7 by volume) + 2 wt.% FEC	45 °C, 1 C, 400 cycles, capacity retention 82%	[98]
Pouch‐type 1000 mAh NCM811/graphite (— V)	1 m LiPF_6_ FEC/AN (7/3 by volume)	RT, 6 C/1 C, 100 cycles, capacity retention 85%	[99]
Coin‐type NCM811/graphite (3–4.4 V) 2 mAh cm^−2^	1 m LiPF_6_ FEC/AN (7/3 by volume)	RT, 4 C/0.3 C (170 mAh g^−1^), 100 cycles, capacity retention —%	[100]
Pouch‐type 1 Ah NCM811/graphite (2.5–4.2 V)	3 m LiPF_6_ EA/FEC (9/1 by volume)	−20 °C, 0.2 C, 1400 cycles, capacity retention 100%; −20 °C, 0.5 C, 4000 cycles, a low capacity decay rate of 0.0097% per cycle; RT, 2 C/1 C, 300 cycles, capacity retention 100%	[101]
Pouch‐type 240 mAh NCM811/graphite (2.8–4.3 V)	LiPF_6_/MDFA/PFPN/FEC (1/7/0.5/1 by molar)	RT, 1 C, 500 cycles, capacity retention 81.8%;	[102]
Pouch‐type 1 Ah SC‐NCM811/graphite (3–4.3 V)	1 m LiPF_6_ 0.02 m LiDFOB FEC/HFE/FEMC (2/2/6 by volume)	RT, 1/3 C, 200 cycles, capacity retention 110.12%; Increase T_2_ by 12.5 °C	[103]
Pouch‐type 1 Ah NCM811/graphite (3–4.2 V)	1 m LiPF_6_ FEC/HFE/FEMC (2/2/6 by volume)	RT, 1/3 C, 100 cycles, capacity retention ≈100%; Increase T_2_ by 17.7 °C	[104]
Pouch‐type 1.0 Ah NCM811/SiMP (2.8–4.3 V)	FEMC‐LHCE+2%FEC	RT, 0.2 C, 200 cycles, capacity retention 62%;	[105]
Coin‐type NCM811/graphite (2.8–4.6 V) 9.5 mg cm^−2^	1 m LiPF_6_ + 0.2 m LiDFOB FEC/EMC/TFA (1/3/1 by volume).	RT, 0.5 C, 100 cycles, capacity retention 83.3%	[106]
Pouch‐type NMA90/graphite (2.5–4.2 V)	1.5 m LiPF_6_ EMC containing 20 wt.% FEC and 1 wt.% LiTDI	RT, 0.5 C/1 C, 500 cycles, capacity retention 80%	[107]
Pouch‐type 10 Ah NCM811/graphite	0.8 m LiFSI‐0.1 m LiTFSI‐0.6 m LiPF_6_ EMC	Increase T_2_ by 67°C	[108]
Pouch‐type 1 Ah NCM811/graphite (3–4.5 V)	0.8 m LiFSI‐0.1 m LiTFSI‐0.6 M LiPF_6_ EMC	RT, 1/3 C, 200 cycles, capacity retention 82.1%	[108]
Pouch‐type LiNi_0.94_Co_0.06_O_2_/graphite (2.7–4.15 V) ≈1 mAh cm^−2^	1.5 M LiPF_6_ EMC + 3 wt.% VC	45°C, 1 C, 500 cycles, capacity retention 81%	[109]
Coin‐type NCM811/MCMB (2.8–4.2 V) 6 mg cm^−2^	1.6 m LiFSI TEOSCN	60°C, 1 C/2 C, 507 cycles, capacity retention 80%	[110]
Pouch‐type 1 Ah NCM811/MCMB (2.8–4.3 V)	1.6 m LiFSI TEOSCN	25°C, 0.2 C, 500 cycles, capacity retention 95%	[110]
Coin‐type NCM811/MCMB (2.8–4.3 V) 6.6 mg cm^−2^	0.8 M LiDFOB + 0.2 m LiBF_4_ SL/FB (2.8/1, by volume)	60°C, 0.5 C, 417 cycles, capacity retention 80%	[111]
Coin‐type NCM811/graphite (2.5–4.5 V) 2.5 mAh cm^−2^	1 m LiTFSI MDFA/MDFSA/TTE (4/1/5 by volume)	25°C, 0.5 C, 400 cycles, capacity retention 80.1%	[112]
Pouch‐type NCM811/graphite (2.5–4.5 V) 2.5 mAh cm^−2^	1 m LiTFSI MDFA/MDFSA/TTE (4/1/5 by volume)	−30°C, 0.2 C, 360 cycles, capacity retention ≈80%	[113]
Coin‐type NCM811/graphite (2.8–4.6 V) 1.5 mAh cm^−2^	1.9 m LiFSI TTMS/TM (1/2 by volume)	RT, 1 C/2 C, 2000 cycles, capacity retention 85%	[114]
Pouch‐type 1 Ah NCM811/graphite (3–4.6 V)	1.9 m LiFSI TTMS/TM (1/2 by volume)	RT, 0.5 C/1 C, 1000 cycles, capacity retention 83%	[114]
Coin‐type NCM811/graphite (2.8–4.3 V) 1.5 mAh cm^−2^	LiFSI/TEP/EC/BTFE (1/1/0.3/3 by molar)	RT, 0.5 C/1 C, 300 cycles, capacity retention 85.4%	[118]
Coin‐type NCM811/graphite (2.5–4.4 V) 1.45 mAh cm^−2^	LiFSI/DME/TTE/FEC (1.0/1.1/3.0/0.2 by molar)	RT, 0.333 C/1 C, 500 cycles, capacity retention 86.8%	[119]
Coin‐type NCM811/graphite (2.5–4.4 V) 2.8 mAh cm^−2^	1.4 m LiFSI DMC/EC/TTE (2/0.2/3 by molar)	25°C, C/3, 600 cycles, capacity retention 94.2%; 25°C, C/3, 100 cycles, capacity retention 94.9%	[120]
Coin‐type LiNi_0.96_Mg_0.02_Ti_0.02_O_2_/graphite (2.5–4.4 V) 1.5 mAh cm^−2^	1.54 m LiFSI DME/FEC/TTE (4.67/1.00/30.84 by weight)	25°C, 0.333 C/1 C, 500 cycles, capacity retention 95.3%	[121]
Coin‐type LiNi_0.96_Mg_0.02_Ti_0.02_O_2_/graphite (2.5–4.4 V) 1.5 mAh cm^−2^	LiFSI/DMC/EC/TTE (1/2/0.2/3 by molar)	25°C, C/3, 200 cycles, capacity retention 97.2%; 60°C, C/3, 100 cycles, capacity retention 80.9%	[122]
Coin‐type NCM811/graphite (2.5–4.4 V) 1.66 mAh cm^−2^	1.44 m LiFSI TMP/FEC/TTE (1.2/0.2/3.0 by molar)	25°C, 0.333 C/1 C, 500 cycles, capacity retention 85.4%	[123]
Coin‐type NCM811/MCMB (2.7–4.3 V) 4.6 ± 0.2 mg cm^−2^	2 m LiTFSI + 2 m LiDFOB TMP/GBL (1/1 by volume)	RT, 0.5 C, 200 cycles, capacity retention 76.8%	[124]
Coin‐type NCA/MCMB (2.7–4.3 V) 4.6 ± 0.2 mg cm^−2^	2 m LiTFSI + 2 m LiDFOB TMP/GBL (1/1 by volume)	RT, 0.5 C, 400 cycles, capacity retention 81.9%	[124]

**Figure 9:**
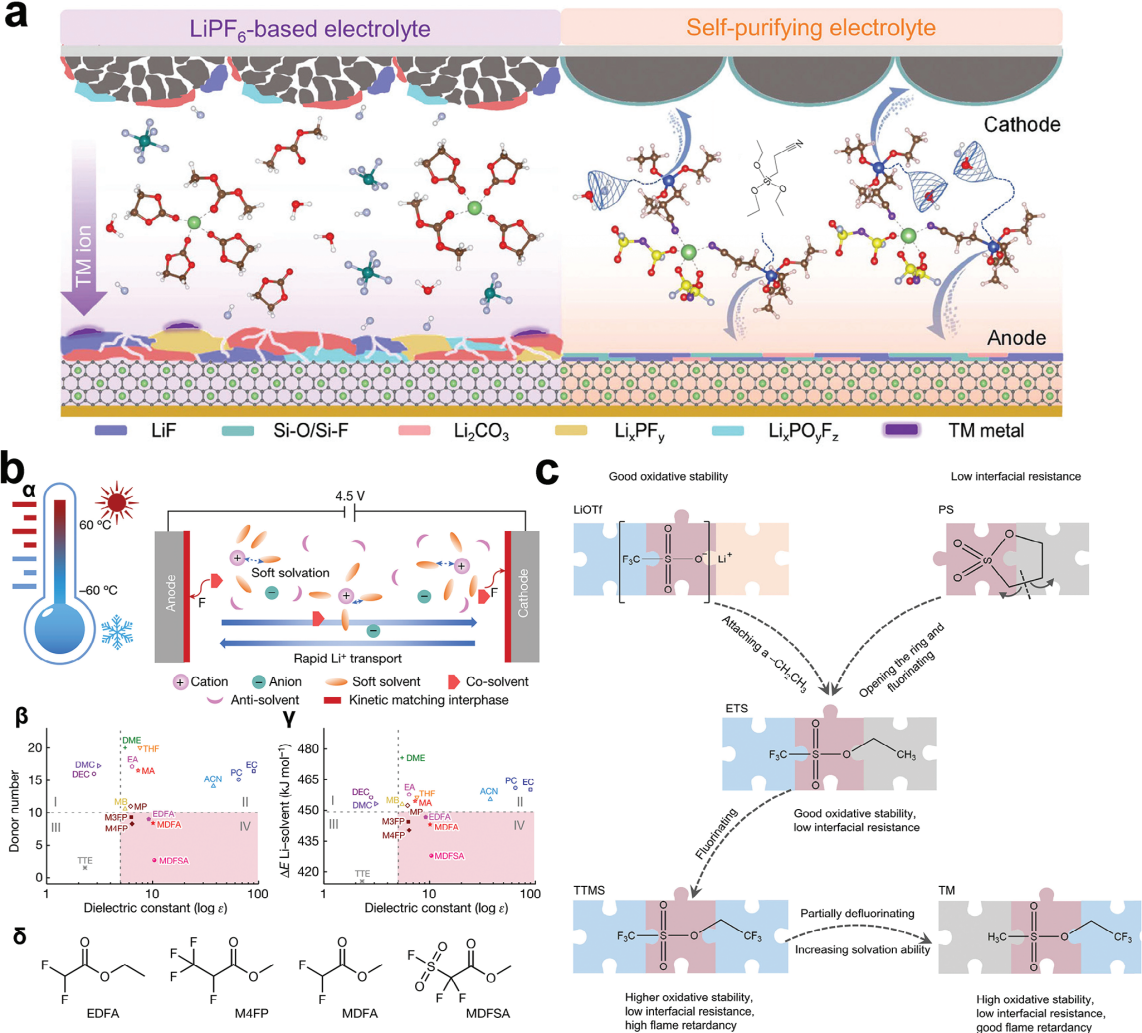
a) Schematic demonstration of the self‐purifying feature and efficient SEI/CEI forming capability of LiFSI‐TEOSCN electrolyte (green: Li atom, red: O atom, yellow: S atom, purple: N atom, silver: F atom, blue: Si atom, green‐black: P atom, tan: C atom, white: H atom). Reproduced with permission.^[^ ^110^ ^]^ Copyright 2022, Royal Society of Chemistry. b) Electrolyte design strategies under extreme operating conditions. *α*, Illustration of the soft solvation between the soft solvent and Li ions, rapid Li‐ion transport, and wide‐temperature range (±60 °C) stability. *β*, The solvent diagram of DN versus dielectric constant. Solvents located in zone IV are denoted as soft solvents, in which the lower DN and higher dielectric constant effectively reduce the Li^+^–solvent affinity without sacrificing kinetic transportation. *γ*, The Li^+^–solvent binding energy from DFT calculations versus experimental dielectric constant. ACN, acetonitrile; DMC, dimethyl carbonate; DME, dimethoxyethane; EA, ethyl acetate; EC, ethylene carbonate; MB, methyl butyrate; MP, methyl propionate; PC, propylene carbonate; THF, tetrahydrofuran. δ, Chemical structure of the soft solvating solvents. Reproduced with permission.^[^ ^113^ ^]^ Copyright 2023, Springer Nature. c) Design processes for multifunctional sulfonate‐based solvents based on the stable LiOTf salt and the PS film‐forming additive. Reproduced under terms of the CC‐BY license. Reproduced with permission.^[^ ^114^ ^]^ Copyright 2023, Springer Nature.

Another class of novel electrolytes is formulated by non‐LiPF_6_ salts (such as LiFSI, LiTFSI, and LiDFOB) and high‐voltage stable solvents with different functional groups (Table 2).^[^ ^110^, ^111^, ^112^, ^113^, ^114^ ^]^ Fan et al. formulate a novel electrolyte consisting of 1.6 m LiFSI and (2‐cyanoethyl)triethoxysilane (TEOSCN), possessing a self‐purifying feature by scavenging unfavorable H^+^ containing species (such as HF and H_2_O) and plus the efficient SEI/CEI forming capability (**Figure** **9**a).^[^ ^110^ ^]^ As a result, the as‐fabricated self‐purifying electrolyte enables NCM811/MCMB full cells with an ultrahigh capacity retention of 81% at 60 °C. Recently, Wang's group reported an electrolyte design principle for high‐Ni LIBs operating under extreme conditions.^[^ ^113^ ^]^ The core principle is selecting solvents with a relatively low donor number (DN) (<10) and high dielectric constant (>5) values, which minimize the Li^+^‐solvent binding energy while still dissociating the lithium salt (Figure 9b). This design strategy enables electrolytes with high ionic conductivity and low Li^+^ desolvation energy (low interfacial resistance) at a wide temperature range. Simultaneously, electrolyte fluorination imparts non‐flammable characteristics to it. As a proof, 1 M LiTFSI is dissolved into MDFA, methyl 2,2‐difluoro‐2 (fluorosulfonyl)acetate (MDFSA), and 1,1,2,2‐ tetrafluoroethyl‐2,2,3,3‐tetrafluoropropylether (TTE). The rationally designed electrolyte enables the 4.5 V NCM811/graphite full cells (2.5 mAh cm^−2^) having the capability of XFC (<15 min), stable cycling over a wide temperature range (−60 to + 60 °C), and non‐flammability. Very recently, Fan et al. also created a novel electrolyte of 1.9 m LiFSI in a mixture of 2,2,2‐trifluoroethyl trifluoromethanesulfonate (TTMS) and 2,2,2‐trifluoroethyl methanesulfonate (TM) (1:2 by volume) (Figure 9c).^[^ ^114^ ^]^ The novel solvents of TTMS and TM integrate the merits of Lithium trifluoromethanesulfonate (LiOTf) and 1,3‐propane sultone (PS). Thus, the as‐formulated electrolyte successfully alleviates the parasitic reactions on the high‐voltage NCM811 cathode and promotes the formation of low impedance SEI layer containing S species (Li_2_SO_x_) on the graphite anode. This electrolyte endows 4.6 V NCM811/graphite (1 Ah) with a high‐capacity retention of 83% after 1000 cycles.

### 3.2 HCEs and LHCEs for High‐Ni LIBs

**Figure 10:**
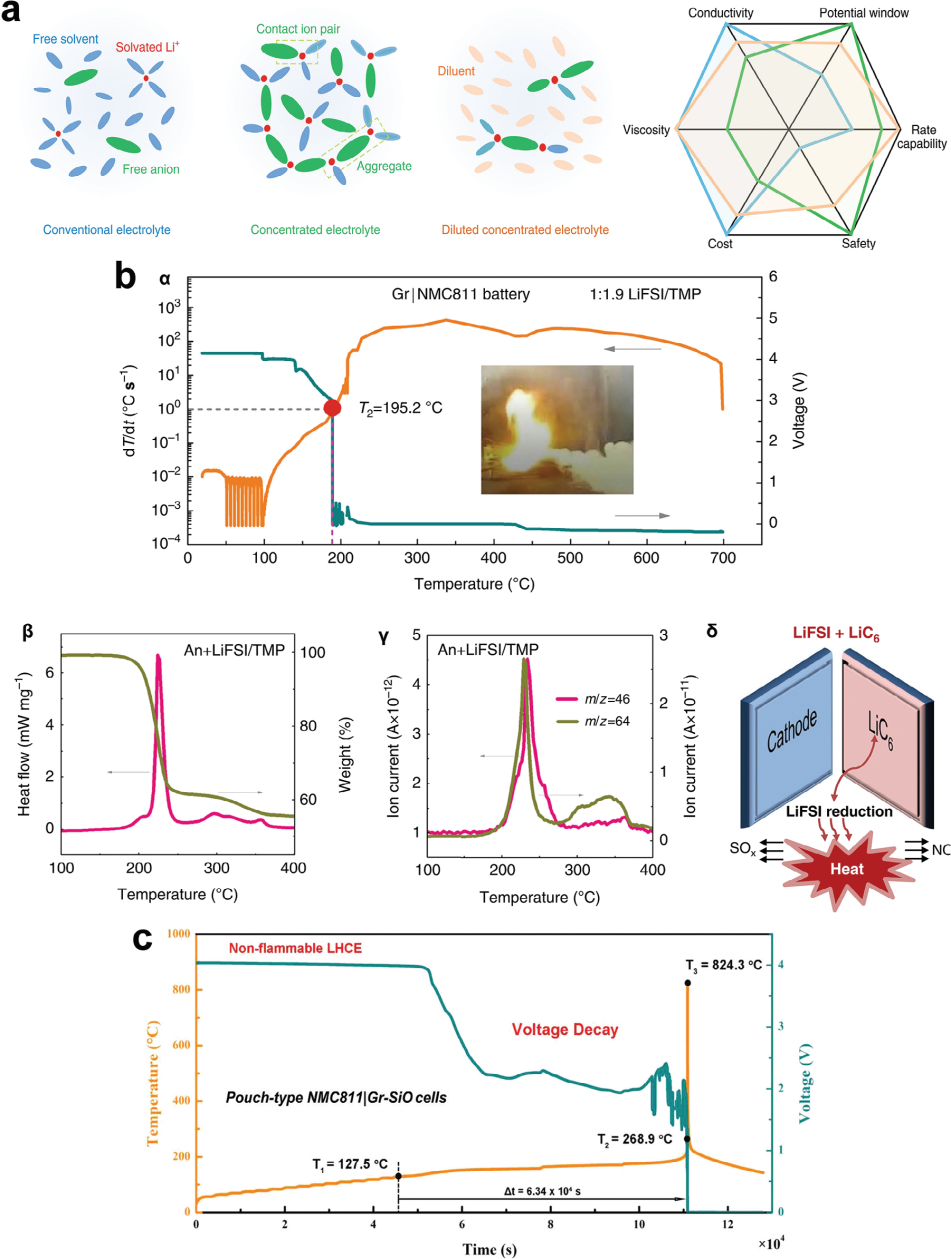
a) Schematic solvation structures of the conventional dilute electrolyte, HCEs, and LHCEs. Reproduced with permission.^[^ ^116^ ^]^ Copyright 2019, Springer Nature. b) Thermal runaway of NCM811/graphite battery with a non‐flammable HCE of LiFSI/TMP. *α*, The dT/dt (ARC testing) of NCM811/graphite battery using an HCE of LiFSI/TMP. The inset demonstrates the battery combustibility under thermal abuse. *β*, DSC trace, and TGA curve of the Anode+LiFSI/TMP sample. *γ*, NO_2_ (m/z = 46) and SO_2_ (m/z = 64) evolution of the Anode + LiFSI/TMP sample. *δ*, Illustration of the proposed thermal runaway mechanism. Reproduced with permission.^[^ ^125^ ^]^ Copyright 2020, Springer Nature. c) ARC‐tested safety features of charged Ah‐level NCM811/Graphite‐SiO pouch cells using nonflammable LHCE (1.0 m LiFSI/FECTEP‐BTFE). Reproduced with permission.^[^ ^127^ ^]^ Copyright 2022, Wiley‐VCH.

When the lithium salt (e.g., LiFSI and LiTFSI) concentration in electrolyte increases to high levels exceeding 3 m, the ratio of contact ion pairs (CIPs) and aggregates (AGGs) increases, while the number of free solvent molecules is drastically decreased (**Figure** **10**a).^[^ ^116^ ^]^ As a result, the HCEs exhibit excellent interface compatibility and flame retardant. However, because of their intrinsic high viscosity and poor wettability with conventional polyolefin separators, the practical applications of HCEs in commercial LIBs are greatly limited. To address this obstacle, low‐polarity dilute solvents (normally hydrofluoroether, such as TTE, and bis(2,2,2‐trifluoroethyl) ether (BTFE)) are added into HCEs to prepare LHCEs.^[^ ^117^ ^]^ The addition of hydrofluoroethers into HCEs does not break the solvation structure of HCEs, but interestingly, the electrolyte viscosity will be greatly decreased, and the ionic conductivity of electrolytes will be enhanced. At present, a series of LHCEs have been designed for performance enhancement of high‐Ni LIBs.^[^ ^116^, ^117^, ^118^, ^119^, ^120^, ^121^, ^122^, ^123^, ^124^, ^125^, ^126^, ^127^ ^]^ The electrochemical compatibility of ether solvents (such as 1,2‐dimethoxyethane (DME)^[^ ^119^, ^121^ ^]^) and phosphate solvents (e.g., triethyl phosphate (TEP)^[^ ^118^, ^127^ ^]^ and trimethyl phosphate (TMP)^[^ ^123^, ^124^, ^125^ ^]^) with both high‐Ni cathode and graphite‐based anodes will be dramatically increased when formulating HCEs and LHCEs. However, NCM811/graphite battery with a nonflammable HCE of LiFSI/TMP still undergoes severe thermal runaway during ARC testing (Figure 10b).^[^ ^125^ ^]^ It is revealed that the considerable heat generated by the reaction of between LiFSI salt and fully lithiated graphite anode (LiC_6_) triggers the thermal runaway of the NCM811/graphite battery. In another case, during ARC testing, NCM811/graphite‐SiO pouch cells with nonflammable a LHCE (1.0 m LiFSI/FEC:TEP:BTFE = 10:20:70 by volume) present a unique self‐discharging behavior, and *T*_2_ is increased by 47.3 °C (Figure 10c).^[^ ^127^ ^]^

In summary of this part, considering the limited oxidative stability of highly flammable LiPF_6_‐carboante electrolytes, it is necessary to develop high voltage resistance and high safety electrolytes for high‐Ni LIBs:

1) First of all, it is very necessary to decipher the oxidative decomposition pathways of conventional carbonate solvents on high‐Ni cathodes (Ni ≥ 80%). New measurement methods, such as in situ differential electrochemical mass spectrometry (DEMS) system, will be very helpful for identifying representative gas byproducts (CO_2_, H_2_, etc.) during LIBs cycles. More importantly, further development of advanced mass spectrometry and spectroscopic approaches (especially in situ methods) to reveal the sources of gas byproducts identifies the key weak points of conventional carbonate solvents. This will provide more guidance for the rational design of the new carbonate solvents (such as fluorinated carbonate solvents) and enhance the cycle performance of the LIBs.

2) The innovations of non‐carbonate novel solvents have achieved great progress recently. Also, the structure screening of non‐carbonate novel solvents requires high throughput computing and experimenting methods. The novel electrolyte compatibility with high‐Ni LIBs must comprehensively consider the solvation structure regulation, oxidative/reductive stability, forming ability of robust interface layer, flame‐retardant, thermal safety evaluation, etc.

3) The present main lithium conducting salts (such as LiPF_6_, LiBOB, LiDFOB, LiTFSI, and LiFSI) in electrolytes are still not satisfactory when used in high‐Ni LIBs. Therefore, except the solvent innovation, designing, synthesizing, and evaluating novel lithium salts is also very important to enhance the performances of high‐Ni LIBs. The following requirements need to be satisfied when developing novel lithium salts: 1) contributing to the formation of stable and robust CEI and SEI layers; 2) thermal stability, non‐toxic and remains stable against oxidative decomposition at the cathode; 3) inert to electrolyte solvents and other cell components such as the collector.

## 4 The Understanding of Thermal Runaway Mechanisms for High‐Ni LIBs

**Figure 11:**
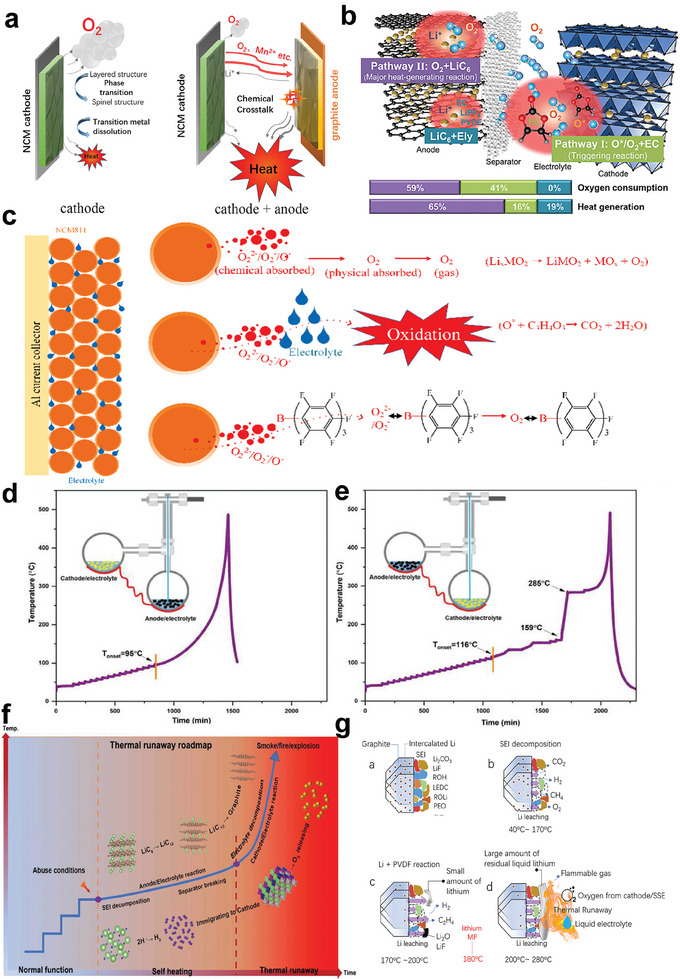
Thermal runaway mechanisms of high‐Ni LIBs. a) Schematic demonstration of proposed chemical crosstalk process between cathode and anode. Reproduced with permission.^[^ ^132^ ^]^ Copyright 2018, Elsevier. b) Proposed mechanisms of cathode‐released oxygen (pathway I: O^∗^/O_2_+EC; pathway II: O_2_+LiC_6_) in triggering the thermal runaway of NCM811/graphite pouch cells. Reproduced with permission.^[^ ^133^ ^]^ Copyright 2021, Elsevier. c) Hazards of highly reactive oxygen generated from NCM811 cathode. Reproduced with permission.^[^ ^134^ ^]^ Copyright 2021, Elsevier. d) The effect of cathode thermal runaway on the thermal stability of anode. e) The effect of anode thermal runaway on the thermal stability of the cathode. f) Thermal runaway route map related to the LiH‐induced heat generation and the H_2_ migration to the cathode. Reproduced with permission.^[^ ^136^ ^]^ Copyright 2021, Wiley‐VCH. g) The thermal degradation pathways of lithiated graphite anode at varied temperatures. Reproduced with permission.^[^ ^137^ ^]^ Copyright 2021, Springer Nature.

Compared to conventional LiFePO_4_ and low‐Ni LIBs, high‐Ni LIBs always suffer from much more severe thermal runaway under abuse conditions (such as thermal, mechanical, and electrical abuse conditions).^[^ ^128^, ^129^, ^130^, ^131^ ^]^ As aforementioned, electrolyte engineering plays a critical role in enhancing the safety of high LIBs. However, the present understandings on the thermal runaway mechanisms of high‐Ni LIBs is still not comprehensive.^[^ ^128^, ^129^, ^130^, ^131^, ^132^, ^133^, ^134^, ^135^, ^136^, ^137^ ^]^ Which electrode dominates the thermal runaway triggering is embroiled into great controversy. Differential scanning calorimetry (DSC) and ARC characterizations are usually applied to study the thermal runaway mechanisms of LIBs.^[^ ^128^ ^]^ In recent years, the role of thermal‐induced cross‐talking effects between cathode and anode in triggering the thermal runaway of high‐Ni LIBs has aroused great interest. It is initially proposed by M. Ouyang et al. that the chemical crosstalk of cathode‐released oxygen to fully lithiated graphite anode will lead to a great heat generation, triggering the thermal runaway of NCM523/graphite battery (**Figure** **11**a).^[^ ^132^ ^]^ Later in NCM811/graphite pouch cells, it is confirmed that two oxygen‐involved endogenous pathways trigger the battery thermal runaway (Figure 11b)^[^ ^133^ ^]^: The reaction between thermal‐indued oxygen with EC solvent is the triggering step, while the reaction between the residue thermal‐indued oxygen and fully lithiated graphite anode brings the battery to an uncontrollable thermal runaway state. In another case, the reaction between oxygen species (O_2_, O_2_^−^, O^−^, etc.) and electrolyte is proposed as the triggering factor of NCM811/graphite battery thermal runaway (Figure 11c).^[^ ^134^ ^]^ In these three reports,^[^ ^132^, ^133^, ^134^ ^]^ it is pointed out that the thermal‐induced release of highly reactive oxygen and their chemical crosstalk contribute to the thermal runaway triggering of high‐Ni LIBs. Differently, N. E. Galushkin et al. suggest that the powerful exothermic reaction related to the recombination of atomic hydrogen accumulated in graphite anode is the thermal runaway triggering factor of 18 650 NCM523/graphite cells.^[^ ^135^ ^]^ A two‐bomb chamber ARC testing system is delicately designed to study the crosstalk of thermal‐induced gas between the cathode and anode.^[^ ^136^ ^]^ It is revealed that the thermal runaway of fully lithiated graphite anode has a greater effect on the cathode thermal runaway (Figure 11d,e). After comprehensive characterizations, it is innovatively proposed that the LiH^[^ ^136^, ^138^, ^139^ ^]^ induced exothermic reactions at the anode side and H_2_ migration to the cathode side is the rooted thermal runaway trigger of NCM523/graphite pouch cell (Figure 11f). K. Amine et al. point out that the thermal‐induced metallic lithium leaching from lithiated graphite anode and H_2_ generation/migration contribute a lot to the thermal runaway of LIBs (Figure 11g).^[^ ^137^ ^]^ Despite in dispute, the consensus is that thermal‐induced cross‐talking effects between the cathode and anode definitely contribute to trigger the thermal runaway of high‐Ni LIBs. As for electrolyte engineering elaborated in this review, we must give full consideration of both interface compatibility and safety enhancement in designing novel additives, solvents, and salts for high‐Ni LIBs.

## 5 Summary and Outlook

As aforementioned (Schemes 1 and 2; Tables 1 and 2), electrolyte engineering for next‐generation high‐Ni (Ni≥80%) LIBs (full cells) with aggressive chemistry has achieved great progress. However, the large‐scale application of high‐Ni LIBs still faces many challenges. The fundamental reason for the poor cycling performance and thermal safety of LIBs using conventional LiPF_6_‐carbonate‐based electrolytes is the inability of the electrolyte to form a robust and stable interfacial layer on the electrode surface. Thus, the electrolytes will continuously react with the delithiated high‐Ni cathode and lithiated anode during long cycles or under abuse conditions. A good electrolyte will form a uniform and dense SEI/CEI layer in situ on the electrode surface during the initial activation process to suppress further reactions. As mentioned by Balbuena et al.,^[^ ^140^ ^]^ the stability of the electrode electrolyte interface layers is more essential than that of the electrolyte components. In accordance with this point, the following critical analysis and perspective will be provided to facilitate the evolution of electrolyte engineering strategies toward practical applications:

1) As for the development of functional additives for conventional LiPF_6_‐carbonate‐based electrolytes, not only the H_2_O/HF inhibiting and scavenging ability but also the forming ability of robust stable interface layer are required to be considered. Obviously, the synergistic effects achieved by incorporating multiple functional motif groups into a single additive or adding several functional additives into an electrolyte are very important to significantly enhance the cycling performances of high‐Ni LIBs. However, there are still several issues to be resolved:
*α*, The working mechanisms of different functional additives or different functional motif groups are still not comprehensive. In the future, their detailed working mechanisms must be clarified based on the detailed characterization on varied interface byproducts (such as solid‐state byproducts, gas byproducts, and soluble byproducts, etc.). Also, it is necessary to depict the decomposition pathways of the functional additive itself on both high‐Ni cathode and anode. In addition, the effects of functional additives or functional motif groups on the electrolyte oxidative/reductive pathways require clarification. Moreover, the effects of functional additives on the thermal stability of electrodes and the thermal safety of large‐format High‐Ni LIBs should be evaluated.
*β*, The structure screening of functional additives is still mainly relies on expensive and low‐efficient trial‐and‐error experiments. In the future, based on the understanding of the aggressive chemistries in high‐Ni LIBs and the working mechanisms of confirmed functional additives, it is necessary to adopt high throughput computing and experimenting methods to design and synthesize a series of potentially high effective functional additives containing different functional motif groups. Also, the synthesis methods of functional additives are required to be feasible, low‐cost, and environmentally friendly. It is also noted here that the design, synthesis, and evaluation of novel functional additives requires the cooperation of researchers who are experienced and knowledgeable in the different fields of theoretical chemistry, organic synthesis, and battery research.
*γ*, It is noted here (Table 1) that the cycling stability of coin‐type high‐Ni LIBs is always poor, while the pouch‐type high‐Ni LIBs exhibit much superior cycle life under the same conditions (e.g., same LiPF_6_‐carboante electrolyte and areal capacity). Additionally, it can be noted that there is a significant difference in the areal capacity of high‐Ni cathodes (from 4.6 to 22 mg cm^−2^) used in different reports. It is hard to demonstrate the reliability of functional additives in highly aggressive chemistry conditions just using LIBs with low‐loading cathodes (<6 mg cm^−2^). Therefore, the effectiveness of functional additives must be evaluated in both coin‐type and pouch‐type high‐load (>15 mg cm^−2^) high‐Ni full cells.
2) Considering the limited oxidative stability of highly flammable LiPF_6_‐carboante electrolytes, it is necessary to develop high voltage resistance and high safety electrolytes for high‐Ni LIBs:
*α*, First of all, it is very necessary to decipher the oxidative decomposition pathways of conventional carbonate solvents on high‐Ni cathodes (Ni≥80%). New measurement methods, such as the in‐situ DEMS system, will be very helpful for identifying representative gas byproducts (CO_2_, H_2_, etc.) during LIBs cycles. More importantly, further development of advanced mass spectrometry and spectroscopic approaches (especially in situ methods) to reveal the sources of gas byproducts identifies the key weak points of conventional carbonate solvents. This will provide more guidance for the rational design of the new carbonate solvents (such as fluorinated carbonate solvents) and enhance the cycle performance of the LIBs.
*β*, The innovations of non‐carbonate novel solvents have achieved great progress recently. Also, the structured screening of non‐carbonate novel solvents requires high throughput computing and experimenting methods. The novel electrolyte compatibility with high‐Ni LIBs must comprehensively consider the solvation structure regulation, oxidative/reductive stability, forming ability of robust interface layer, flame‐retardant, thermal safety evaluation, etc.
γ, The present main lithium conducting salts (such as LiPF_6_, LiBOB, LiDFOB, LiTFSI, and LiFSI) in electrolytes are still not satisfactory when used in high‐Ni LIBs. Therefore, except the solvent innovation, designing, synthesizing, and evaluating novel lithium salts is also very important to enhance the performances of high‐Ni LIBs. The following requirements need to be satisfied when developing novel lithium salts: 1) contributing to the formation of stable and robust CEI and SEI layers; 2) thermal stability, non‐toxic and remains stable against oxidative decomposition at the cathode; 3) inert to electrolyte solvents and other cell components such as the collector. For example, to break the obstacles of the serious aluminum collector corrosion that occurred in LiTFSI‐based and LiFSI‐based electrolytes, a non‐corrosive sulfonimide salt lithium((difluoromethanesulfonyl)(trifluoromethanesulfonyl)imide (LiDFTFSI)) was successfully synthesized.^[^ ^141^ ^]^ Recently, K. Liu et al. designed an asymmetric Li salt, lithium 1,1,1‐trifuoro‐N‐[2‐[2‐(2‐methoxyethoxy)ethoxy)]ethyl] methanesulfonamide (LiFEA), that exhibits a large apparent donor number and Li^+^ transference number, dramatically improving the fast‐cycling capability of the NCM811/Li cell.^[^ ^142^ ^]^ Apart from these, a highly fluorinated (8‐CF_3_) aluminum‐centered lithium salt of lithium perfluoropinacolatoaluminate (LiFPA) was reported, which can facilitate the formation of a protective passivating interphase layer on both electrodes.^[^ ^143^ ^]^
3) High‐Ni LIBs always suffer from severe thermal runaway under abuse conditions. However, the present understandings on the thermal runaway mechanisms of high‐Ni LIBs is still not comprehensive. Despite in dispute, the consensus is that thermal‐induced cross‐talking effects between the cathode and anode definitely contribute to trigger the thermal runaway of high‐Ni LIBs. As for electrolyte engineering, we must give full consideration to both interface compatibility and safety enhancement in designing novel additives, solvents, and lithium salts for high‐Ni LIBs. It is noted that in situ solidification/polymerization of electrolytes can potentially increase the thermal safety of high‐Ni LIBs by blocking the thermal‐induced cross‐talking effects.

## Conflict Of Interest

The authors declare no conflict of interest.
